# Comparative effectiveness of immunosuppressive drugs and corticosteroids for lupus nephritis: a systematic review and network meta-analysis

**DOI:** 10.1186/s13643-016-0328-z

**Published:** 2016-09-13

**Authors:** Jasvinder A. Singh, Alomgir Hossain, Ahmed Kotb, George A. Wells

**Affiliations:** 1 Medicine Service, VA Medical Center, Birmingham, AL USA; 2 Department of Medicine at the School of Medicine, and Division of Epidemiology at the School of Public Health, University of Alabama at Birmingham, Faculty Office Tower 805B, 510 20th Street S, Birmingham, AL 35294 USA; 3 Department of Orthopedic Surgery, Mayo Clinic College of Medicine, Rochester, MN USA; 4 Ottawa Heart Institute and the University of Ottawa, Ottawa, ON Canada

**Keywords:** Network meta-analysis, Immunosuppressive drugs, Glucocorticoids, Lupus nephritis, Lupus, Renal remission, Renal relapse, Tacrolimus, Cyclophosphamide, Mycophenolate mofetil

## Abstract

**Background:**

There is a lack of high-quality meta-analyses and network meta-analyses of immunosuppressive drugs for lupus nephritis. Our objective was to assess the comparative benefits and harms of immunosuppressive drugs and corticosteroids in lupus nephritis.

**Methods:**

We conducted a systematic review and network meta-analysis (NMA) of trials of immunosuppressive drugs and corticosteroids in patients with lupus nephritis. We calculated odds ratios (OR) and 95 % credible intervals (CrI).

**Results:**

Sixty-five studies that met inclusion and exclusion criteria; data were analyzed for renal remission/response (37 trials; 2697 patients), renal relapse/flare (13 studies; 1108 patients), amenorrhea/ovarian failure (eight trials; 839 patients) and cytopenia (16 trials; 2257 patients). Cyclophosphamide [CYC] low dose (LD) and CYC high-dose (HD) were less likely than mycophenolate mofetil [MMF] and azathioprine [AZA], CYC LD, CYC HD and plasmapharesis less likely than cyclosporine [CSA] to achieve renal remission/response. Tacrolimus [TAC] was more likely than CYC LD to achieve renal remission/response. MMF and CYC were associated with a lower odds of renal relapse/flare compared to PRED and MMF was associated with a lower rate of renal relapse/flare than AZA. CYC was more likely than MMF and PRED to be associated with amenorrhea/ovarian failure. Compared to MMF, CYC, AZA, CYC LD, and CYC HD were associated with a higher risk of cytopenia.

**Conclusions:**

In this systematic review and NMA, we found important differences between immunosuppressives used for the treatment of lupus nephritis. Patients and physicians can use this information for detailed informed consent in a patient-centered approach. Study limitations of between-study clinical heterogeneity and small sample size with type II error must be considered when interpreting these findings.

**Systematic review registration:**

PROSPERO: CRD42016032965

**Electronic supplementary material:**

The online version of this article (doi:10.1186/s13643-016-0328-z) contains supplementary material, which is available to authorized users.

## Background

Lupus is a chronic autoimmune disease that frequently involves the kidney. Lupus nephritis can lead to kidney failure, dialysis, and even premature death if not treated appropriately. Lupus primarily affects young women and is more common and more severe in racial/ethnic minorities, who experience worse outcomes [1–5].

Comparative effectiveness research (CER) of drugs used to treat lupus nephritis is an imperative [6]. Patients, facing difficult decisions related to their treatment options, such as those related to life- and/or organ-threatening clinical situations (active lupus nephritis, for example) need information about possible harms and benefits of available treatment options in a format that provides comparisons of multiple treatment options.

The 2012 American College of Rheumatology (ACR) lupus nephritis treatment guidelines [7] and the Cochrane systematic review of interventions for lupus nephritis [8] assessed literature up to 2010 and 2012, respectively. However, indirect comparisons were not performed in either. Few lupus nephritis treatments have been compared directly in clinical trials. This leaves a large knowledge gap. Clinicians and patients have to choose between various immunosuppressive drugs in the absence of such knowledge. Therefore, we need evidence synthesis using valid methods to incorporate indirect and direct comparisons of efficacy/harms of these treatments.

A state-of-the-art network meta-analysis (NMA), with updated information, is a necessary precursor to the development of a clinical decision-making tool for physician and their patients with lupus nephritis. This information can be very helpful to patients during the treatment decision-making process for new disease, disease flare or refractory disease. It is not surprising that for a rare condition such as lupus with roughly 161,000 patients in the USA [9], most multicenter trials have <500 patients (often 50–200 patients). This makes many trials underpowered for assessing treatment-related differences in disease outcomes.

One useful approach is the use of composite outcomes, which have been widely used to address important clinical questions in obstetrics, cardiology, and other disciplines [10–12]. Assessments of treatment options using composite outcomes can help answer important question in a timely fashion without requiring studies with large sample sizes. This study aimed to perform a systematic review and NMA to compare benefits and harms of immunosuppressive drugs compared to each other and to corticosteroids focusing on four composite benefit/harm outcomes: (1) renal remission/response; (2) renal relapse/flare; (3) ovarian failure/amenorrhea; and (4) bone marrow toxicity.

## Methods

We used rigorous methods for the systematic review and NMA based on the Agency for Healthcare Research and Quality (AHRQ) recommendations [13], the Cochrane handbook [14], and the PRISMA guidelines. The Institutional Review Board at the University of Alabama at Birmingham (UAB) approved the study. The need for informed consent was waived for this systematic review, since no human subjects were involved. The study protocol was registered in PROSPERO, CRD42016032965 (http://www.crd.york.ac.uk/PROSPERO/).

### Methods for systematic review: study eligibility criteria, outcomes and data abstraction

This systematic review included randomized controlled trials (RCTs) or controlled clinical trials (CCTs) of immunosuppressive drugs or corticosteroids for lupus nephritis, published in English that reported any safety or efficacy outcome. Included medications were: corticosteroids [PRED]; cyclophosphamide [CYC]; mycophenolate mofetil [MMF]; azathioprine [AZA]; cyclosporine [CSA]; tacrolimus [TAC]; or rituximab [RTX], Belimumab studies could not be included in this systematic review since these studies included patients with lupus, and only a small proportion had active lupus nephritis. A Cochrane systematic review of belimumab for lupus in underway [15]. There were no restrictions with regards to the medication dose or the duration of medication use.

Experienced librarians (JJ and TR) updated two systematic reviews [7, 8] from their search end dates (August 2010 and April 2012, respectively) to September 2013 using the PubMed database. The search used the following terms: (Lupus[text word] OR "Lupus Vulgaris"[MeSH] OR "Lupus Erythematosus, Cutaneous"[MeSH] OR "Lupus Erythematosus, Systemic"[Mesh]) AND ("Kidney Diseases"[MeSH] OR nephropath*[text word] OR Transplants[MeSH] OR Transplantation[MesH] OR transplantation[subheading] OR transplant*[text word] OR "Kidney"[Mesh] OR Kidney*[text word] OR Renal*[text word] OR "End Stage Renal Disease"[text word] OR ESRD[text word] OR Glomerulonephr*[text word] OR "GN"[text word] OR "crescentic GN"[text word]) NOT ("animals"[MeSH] NOT "humans"[MeSH]).

Raw data abstracted for the ACR lupus nephritis guidelines systematic review [7] were obtained (courtesy Dr. Jennifer Grossman (JG), see acknowledgment section), or were abstracted from the Revman tables of the Cochrane Systematic Review [8]. A librarian (CH) also performed a search for all lupus trials for harms (for conditions other than lupus nephritis) in PubMed and SCOPUS from inception to February 2014, based on an a priori assumption that treatment-related harms may not depend on whether kidney is involved or not. Examination of data from this search revealed little additive data for harms, but added clinical heterogeneity related to differences in patient population. Therefore, after careful consideration of pros and cons, we decided not to use these data in analyses.

The PICO (patient, intervention, comparator, outcome) for our systematic review and NMA were defined as follows:

P: Adults 18 years or older, meeting the 1987 American College of Rheumatology Classification criteria for systemic lupus erythematosus (SLE) [16], who have lupus nephritis.

I: Immunosuppressant drug alone or in combination with other immunosuppressant drugs or biologics (such as rituximab) or corticosteroids. Medication doses were categorized as low, standard or high dose/duration (LD, SD and HD).

C: Placebo or another immunosuppressive with/without biologic.

O: Benefit and harm outcomes (renal remission/response, renal relapse/flare, fertility, bone marrow suppression), defined as follows.

Benefits were assessed by two composite outcomes (for detailed definitions of components, see Additional file 1): (1) renal remission/response (indicating success of therapy): included complete renal remission, [17] partial renal remission [18, 19] and renal response; (2) renal relapse/flare (indicating failure of therapy): included renal relapse [20] and renal flare (Additional file 1). Harms were assessed by two composite outcomes: (1) ovarian failure/amenorrhea: included ovarian failure and amenorrhea; and (2) bone marrow toxicity: cytopenia including leucopenia.

Two trained abstractors (AO, AB) independently reviewed abstracts and titles, abstracted data in duplicate directly into Microsoft excel sheets and assessed the risk of bias according to the Cochrane risk of bias tool [21]. We examined the following domains as low or high risk of bias or unclear risk (lack of information or uncertainty about potential for bias): randomization sequence generation, allocation sequence concealment, blinding of participants, personnel and outcome assessors, incomplete outcome data (primary outcome data reporting, dropout rates and reasons for withdrawal, appropriate imputation of missing data, an overall completion rate ≥80 %), and selective outcome reporting and other potential threats to validity (considering external validity, e.g., relevant use of co-interventions, bias due to funding source). An adjudicator (JS) resolved any disagreements not resolved by consensus. An expert rheumatologist (JS) and an expert in lupus (JG) examined for similarity of studies prior to performing evidence synthesis by the examination of similarity of study population and interventions.

We designated doses as follows: (1) CYC: standard-dose/duration , SD: 0.5–1.0 gm/m^2^ intravenously (IV) q month for 6–12 months or 2–2.5 mg/kg orally (PO) daily × 3–6 months; high-dose/duration, HD: dose higher or duration longer than SD; low-dose/duration, LD: lower dose or duration shorter than SD, including the EURO-lupus dose, 500 mg IV q14 days × 6 doses (mean 3 g); (2) AZA: SD, 1–3 mg/kg po daily; HD, >3 mg/kg po daily; (3) LEF: 1 mg/kg po qd × 3 days then 30 mg po qd x 6 months; and (4) PRED: SD, prednisone/methylprednisolone 1 gm/m^2^ IV q month × 6 months or prednisone 60 mg po qd 1–3 months then tapered over 3–12 months as tolerated; HD, prednisone/methylprednisolone 1gm/m^2^ qd IV × 3, then one dose q month for 1 year or prednisone 1 mg/kg daily for 4–8 weeks (or unspecified period). When dose is not specified, medication dose is the standard dose.

### Bayesian network meta-analysis (NMA)

We used Bayesian mixed treatment comparison (MTC) meta-analyses [22–24] to assess the comparative effectiveness of one immunosuppressive drug vs. another and immunosuppressive drugs vs. corticosteroids. Bayesian MTC meta-analysis using a binomial likelihood model was conducted using WinBUGS software (MRC Biostatistics Unit, Cambridge, UK) which allows inclusion of data from multi-arm trials [25, 26] We conduced random-effects NMA and assessed model fit and the choice of model (random vs. fixed effects) based on the assessment of the deviance information criterion (DIC) and the comparison of residual deviance to the number of unconstrained data points [25, 27].

We assigned vague priors, such as N(0, 100^2^) for basic parameters throughout [25] and informative priors for the variance parameter based on Turner et al. [28]. We evaluated the model diagnostics including trace plots and the Brooks-Gelman-Rubin statistic to ensure model convergence [25, 29]. We fit three chains in WinBUGS for each analysis, with 40,000 iterations, and a burn-in of 40,000 iterations [29, 30] Both MTC and traditional meta-analysis require studies to be sufficiently similar in order to pool their results. We investigated heterogeneity, where warranted, with subgroup analyses and meta-regressions [26, 31]. We examined consistency-inconsistency plots for evidence of inconsistency, and chose the appropriate model for our analyses. We obtained point estimates using odds ratios (OR) and 95 % credible intervals (CrI) using Markov Chain Monte Carlo (MCMC) methods. Transformation of the OR to relative risk (RR) and risk difference was done to allow ease for interpretation for clinicians and patients. The quality of evidence was assessed as recommended in a recent study [32].

Sensitivity analysis was performed by limiting analyses to partial/complete remission rather than combining this with renal response for the composite renal remission/response. We constructed staircase diagrams, another pictorial way to see comparisons of various treatments to each other. Rankograms were constructed to model the probabilities of the treatment rankings, representing the best to the last ranks.

## Results

### Study characteristics

**Fig. 1:**
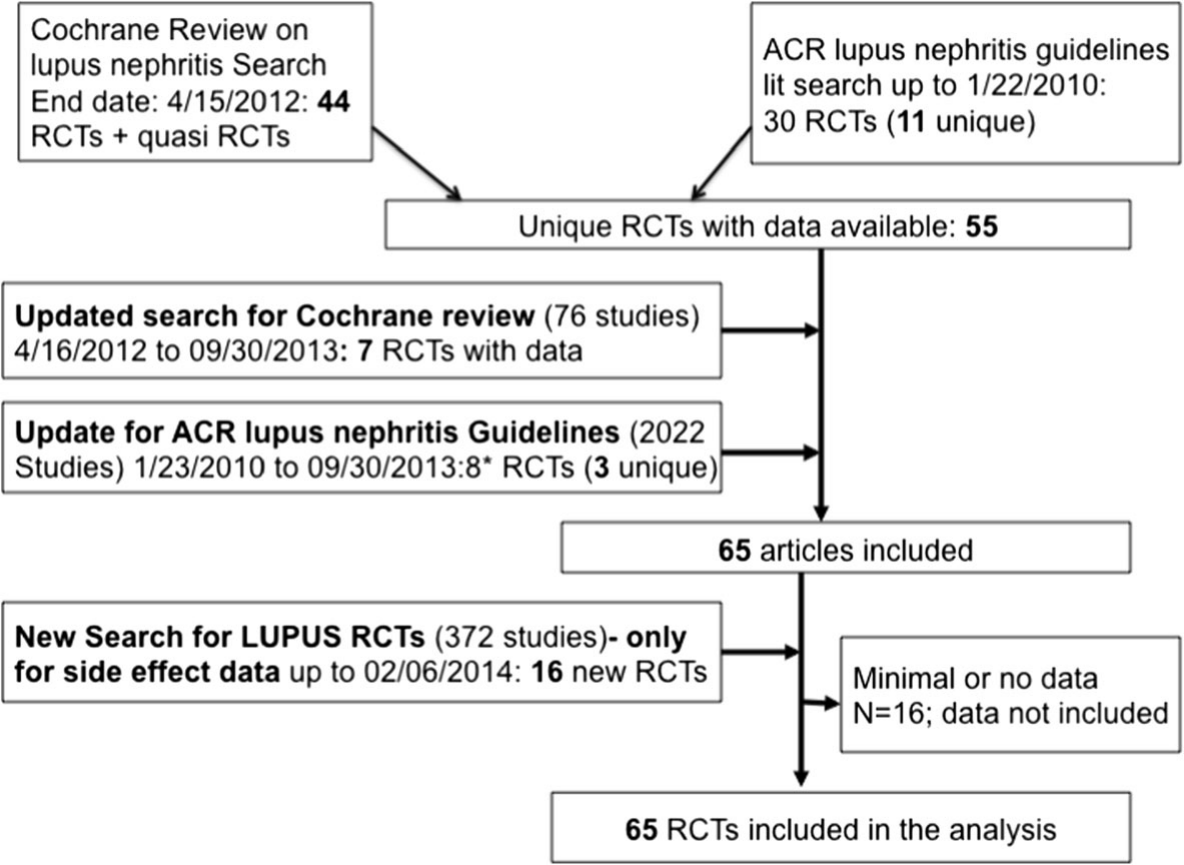
PRISMA study flow chart for study selection. Legend: none

Sixty-five studies met inclusion and exclusion criteria that included CYC, MMF, AZA, calcineurin inhibitors (cyclosporine, tacrolimus), rituximab, corticosteroids, plasmapharesis, or leflunomide (Fig. 1). The Additional file 2 shows the search strategy. An additional file shows the PRISMA checklist (see Additional file 3).

**Fig. 2:**
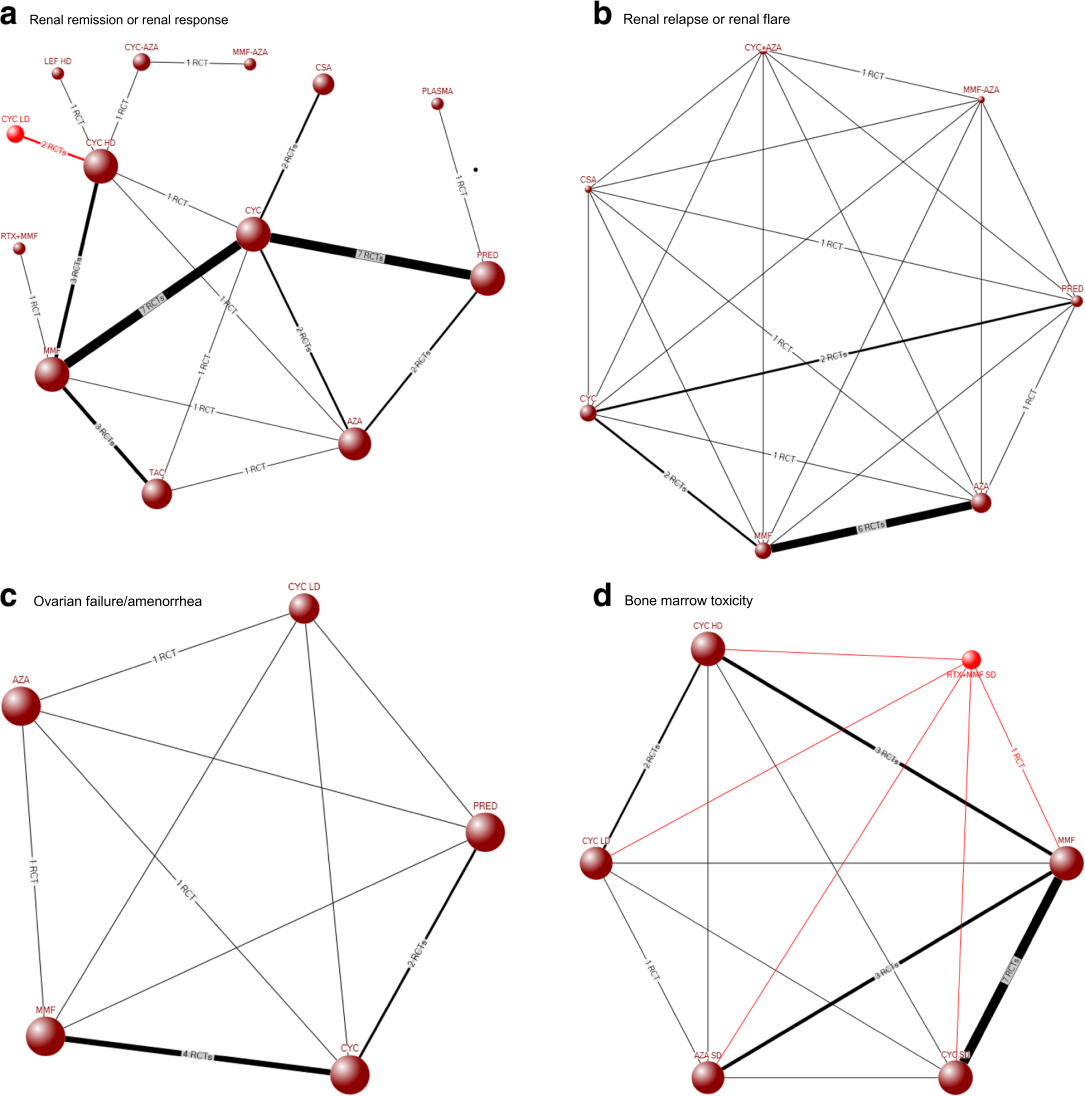
Network diagrams for composite study outcomes in lupus nephritis, renal remission or renal response (**a**), renal relapse or renal flare (**b**), fertility issues (**c**), and bone marrow toxicity (**d**). Legend: Each *node* shows the treatment compared along with the number of RCTs that provide evidence. The size of each node is proportional to the sample size. **a** shows only the direct evidence, while **b**–**d** show both direct and indirect evidence. Direct evidence is indicated by *lines* indicating the number of RCTs providing the evidence and indirect by *lines* without this information. This was done since there were several direct comparator studies available for **a**, and addition of connections based on indirect evidence to **a** would make the network diagram very complex and difficult to visualize and understand

Network diagrams for all outcomes are shown in Fig. 2. Most studies (88 %) compared induction or induction and maintenance regimens. An additional file shows this in more detail (see Additional file 4). The study sample size ranged from 10 to 370. Of these, 32 % of the studies were conducted in the USA and 43 % were multicenter.

A detailed risk of bias using the Cochrane risk of bias tool is provided in Table 1. Randomization was low-risk in 56 %, unclear in 39 % and high-risk in 5 % (Table 1). Most trials were low-risk for blinding of assessor (59 %), blinding of participant (54 %), intention to treat (57 %). On the other hand, only 38 % of trials were low-risk for allocation concealment and it was unclear in 59 %. Although some clinical heterogeneity was detected between trials overall, we did not notice any clinically significant systematic differences in patient populations or disease stages between various medications.

**Table 1:** Risk of bias of included studies according to the Cochrane Risk of Bias tool^a^

Author	Year	Randomization	Allocation concealment	Blinding of assessor and/or physician (for assessment of objective outcomes)	Blinding of participants (for assessment of subjective outcomes)	Intention to treat	Free of selective reporting	Source of funding
Austin	2009	Low risk	Low risk	High risk	High risk	Unclear risk	Unclear risk	Unclear risk
Carette	1983	Unclear risk	Unclear risk	High risk	High risk	Low risk	High risk	Low risk
Steinberg	1991	Low risk	Low risk	Low risk	Low risk	Low risk	Unclear risk	Unclear risk
Donadio	1976	Unclear risk	Unclear risk	High risk	High risk	Low risk	Unclear risk	Low risk
Pohl	1991	Unclear risk	Unclear risk	High risk	High risk	Unclear risk	Unclear risk	Unclear risk
Wang	2007	Unclear risk	Unclear risk	High risk	High risk	High risk	Unclear risk	Unclear risk
Isenberg; An analysis of ALMS study	2010	Low risk	Low risk	Low risk	Unclear risk	Low risk	Low risk	Unclear risk
Appel (ALMS study)	2009	Low risk	Low risk	Low risk	Unclear risk	Low risk	Low risk	Unclear risk
Austin	1986	Low risk	Unclear risk	Unclear risk	Unclear risk	Unclear risk	Unclear risk	Unclear risk
Balletta	1992	Unclear risk	Unclear risk	Low risk	Low risk	Low risk	Unclear risk	Low risk
Bao	2008	Low risk	Low risk	Low risk	Low risk	Low risk	Low risk	Unclear risk
Barron	1982	High risk	High risk	Low risk	Low risk	High risk	High risk	Low risk
Boumpas	1992	Unclear risk	Low risk	Low risk	Low risk	Low risk	Low risk	Unclear risk
Cade	1973	High risk	Unclear risk	Low risk	Low risk	Unclear risk	Unclear risk	Unclear risk
Chan	2000	Low risk	Unclear risk	Low risk	Low risk	Low risk	Low risk	Unclear risk
Chen	2011	Low risk	Low risk	Low risk	Low risk	Low risk	Low risk	Unclear risk
Clark	1981	Unclear risk	Unclear risk	Low risk	Low risk	Low risk	Low risk	Low risk
Clark	1984	Unclear risk	Unclear risk	Low risk	Low risk	Unclear risk	Unclear risk	Unclear risk
Contreras	2002	Low risk	Low risk	Low risk	Low risk	Low risk	Low risk	Unclear risk
CYCLOFA-LUNE Study	2010	Low risk	Low risk	Low risk	Low risk	Low risk	Low risk	Low risk
Derksen	1988	Low risk	Unclear risk	Unclear risk	Unclear risk	Unclear risk	Unclear risk	Unclear risk
Donadio	1974	Low risk	Unclear risk	Low risk	Low risk	Unclear risk	High risk	Low risk
Donadio	1978	Low risk	Unclear risk	Low risk	Low risk	Unclear risk	Unclear risk	Unclear risk
Doria	1994	Unclear risk	Unclear risk	Unclear risk	Unclear risk	Unclear risk	Unclear risk	Unclear risk
Dyadyk	2001	Unclear risk	Unclear risk	Unclear risk	Unclear risk	Unclear risk	Unclear risk	Unclear risk
El-Shafey	2010	Low risk	Unclear risk	Low risk	Low risk	Low risk	Low risk	Low risk
Fu	1998	Low risk	Low risk	Low risk	Low risk	Low risk	High risk	Low risk
Ginzler	2005	Low risk	Low risk	Low risk	Low risk	Low risk	Low risk	Unclear risk
Gourley	1996	Low risk	Low risk	Low risk	Low risk	Low risk	Low risk	Low risk
Grootscholten (Dutch Lupus study)	2006	Low risk	Low risk	Low risk	Low risk	Low risk	Low risk	Unclear risk
Hahn	1975	Low risk	Low risk	Low risk	Low risk	Low risk	Low risk	Low risk
Hong	2007	Unclear risk	Unclear risk	Unclear risk	Unclear risk	Unclear risk	Unclear risk	Unclear risk
Houssiau	2002	Low risk	Unclear risk	Low risk	Low risk	Low risk	Low risk	Low risk
Lewis	1992	Low risk	Unclear risk	Low risk	Low risk	Low risk	Low risk	Low risk
Li	2009a	Low risk	Low risk	Low risk	Low risk	Low risk	Low risk	Unclear risk
Li	2009b	Unclear risk	Unclear risk	Low risk	Low risk	Low risk	Low risk	Low risk
Lui	1997	Unclear risk	Unclear risk	Unclear risk	Unclear risk	Unclear risk	Unclear risk	Unclear risk
LUNAR Study	2012	Low risk	Unclear risk	Low risk	Low risk	Low risk	Low risk	Unclear risk
MAINTAIN Nephritis Study	2010	Low risk	Unclear risk	Low risk	Low risk	Low risk	Low risk	Low risk
Mitwalli	2011	Unclear risk	Unclear risk	Unclear risk	Unclear risk	Unclear risk	Unclear risk	Unclear risk
Mok	2009	Unclear risk	Unclear risk	Unclear risk	Unclear risk	Unclear risk	Unclear risk	Unclear risk
Moroni	2004	Low risk	Low risk	Low risk	Low risk	Low risk	Low risk	Unclear risk
Mulic-Basic	2008	Unclear risk	Unclear risk	Unclear risk	Unclear risk	Unclear risk	Unclear risk	Unclear risk
My-Lupus Study	2010	Unclear risk	Unclear risk	Unclear risk	Unclear risk	Unclear risk	Unclear risk	Unclear risk
Ong	2005	Low risk	Low risk	Low risk	Low risk	Low risk	Low risk	Unclear risk
Sabry	2009	High risk	High risk	Low risk	Low risk	Low risk	Low risk	Low risk
Sesso	1994	Unclear risk	Unclear risk	High risk	Low risk	Low risk	Unclear risk	Low risk
Steinberg	1971	Low risk	Low risk	Low risk	Low risk	Low risk	Low risk	Unclear risk
Sundel/Sandel	2008	Unclear risk	Unclear risk	Unclear risk	Unclear risk	Unclear risk	Unclear risk	Unclear risk
Wallace	1998	Unclear risk	Unclear risk	Unclear risk	Unclear risk	Unclear risk	Unclear risk	Unclear risk
Yee	2004	Low risk	Unclear risk	Low risk	Low risk	Low risk	High risk	Low risk
Li	2012	Unclear risk	Unclear risk	High risk	High risk	High risk	Unclear risk	Low risk
Yap	2012	Unclear risk	Unclear risk	High risk	High risk	High risk	Unclear risk	Unclear risk
Stoenoiu	2012	Unclear risk	Unclear risk	Unclear risk	Unclear risk	High risk	Unclear risk	Unclear risk
Chen	2012	Low risk	Low risk	High risk	High risk	High risk	Unclear risk	Unclear risk
Arends (long term FU of Dutch Lupus study)	2012	Low risk	Low risk	Low risk	Low risk	Low risk	Low risk	Unclear risk
Sundel (report of induction and maintenance phases of ALMS study)	2012	Low risk	Low risk	Low risk	Unclear risk	Low risk	Low risk	Unclear risk
Walsh (post-hoc subgroup analysis of ALMS)	2013	Low risk	Low risk	Low risk	Unclear risk	Low risk	Low risk	Unclear risk
PetrI	2010	Low risk	Unclear	High risk	High risk	Low risk	Unclear risk	Low risk
Zeher	2011	Low risk	Low risk	High risk	High risk	High risk	Unclear risk	High risk
Dooley	2011	Unclear risk	Unclear risk	Unclear risk	Unclear risk	High risk	Unclear risk	High risk

Radhakrishnan 2010 was a pooled analyses of two studies, and Mok 2001, Hu 2002, and Wang 2008 [33–36] were observational studies used in the Cochrane Review; therefore, risk of bias could not be assessed for these studies

^a^Higgins JP, Altman DG, Sterne JA. Chapter 8: Assessing risk of bias in included studies. Cochrane Handbook for Systematic Reviews of Interventions Version 5.1.0 (updated March 2011). The Cochrane Collaboration, 2011. Available from http://handbook.cochrane.org/. In: Higgins JP, Green S, eds 2011

### Treatment efficacy: complete/partial renal remission/response

Thirty-seven trials with 2697 patients provided data for the composite outcome, partial or complete renal remission or renal response (two trials were excluded since they had variable duration of treatments based on response to initial treatment, also associated with high standard errors and wide CrI, leading to problems with convergence of the model when included). There were 34 two-arm and three three-arm trials. Table 2 shows only the significant odds ratios, relative risk and risk differences only, and an additional file shows all comparisons in more detail (see Additional file 5). CYC, MMF, CSA, and TAC were superior to corticosteroids alone in achieving renal remission/response (Table 2). CYC low dose (LD) was less likely than MMF, TAC, CSA, and CYC and CYC HD less likely than MMF and CSA to achieve renal remission/response. CSA was more likely than plasmapharesis and azathioprine to achieve renal remission/response (Table 2). The quality of evidence was rated as moderate (downgraded for imprecision). Absolute event rates ranged from 28 to 75 % and are shown in more detail in an additional file (see Additional file 6).

**Table 2:** Significant differences^a^ between treatments of lupus nephritis for a composite end-point of renal remission or renal response (includes partial remission, complete remission and renal response)

Treatment	Reference	Odds ratio (95 % CrI)	Relative risk (95 % CrI)	Risk difference % (95 % Crl)
CYC	PRED	*2.35 (1.28, 4.23)*	*1.60 (1.60, 2.24)*	*0.21 (0.06, 0.34)*
MMF		*3.26 (1.57, 6.72)*	*1.82 (1.82, 2.60)*	*0.28 (0.11, 0.44)*
TAC		*2.51 (1.11, 5.76)*	*1.64 (1.64, 2.44)*	*0.22 (0.03, 0.41)*
CSA		*5.69 (2.02, 17.61)*	*2.15 (2.15, 3.18)*	*0.40 (0.17, 0.58)*
CYC LD	CYC	*0.32 (0.10, 0.89)*	*0.51 (0.51, 0.95)*	*−0.26 (−0.45, −0.03)*
CYC LD	MMF	*0.23 (0.08, 0.61)*	*0.45 (0.45, 0.81)*	*−0.34 (−0.53, −0.12)*
CYC HD		*0.40 (0.20, 0.74)*	*0.65 (0.65, 0.89)*	*−0.22 (−0.38, −0.07)*
CSA	AZA	*3.20 (1.04, 10.19)*	*1.53 (1.53, 2.39)*	*0.26 (0.01, 0.47)*
CYC LD	TAC	*0.30 (0.09, 0.91)*	*0.50 (0.50, 0.95)*	*−0.28 (−0.50, −0.02)*
PLASMA	CSA	*0.19 (0.04, 0.92)*	*0.49 (0.49, 0.97)*	*−0.38 (−0.66, −0.02)*
CYC LD		*0.13 (0.03, 0.52)*	*0.38 (0.38, 0.76)*	*−0.45 (−0.69, −0.15)*
CYC HD		*0.23 (0.06, 0.72)*	*0.55 (0.55, 0.87)*	*−0.33 (−0.57, −0.08)*
CYC HD		*0.23 (0.06, 0.72)*	*0.55 (0.55, 0.87)*	*−0.33 (−0.57, −0.08)*
Random-effects model	Residual deviance		82.2 vs. 77 data points	
	Deviance information criteria		389.332	
Fixed-effects model	Residual Deviance		88.8 vs. 77 data points	
	Deviance information criteria		390.488	

Based on 37 RCTs with 2697 patients: 35 two-arm trials and three three-arm trials

^a^For all comparisons of treatments of lupus nephritis for renal remission/response please see Appendix 5

For absolute rates for events used for calculation of risk difference, please see Appendix 6

*OR* odds ratio, *RR* relative risk, *RD* risk difference, *CrI* credible interval, *CYC* cyclophosphamide, *MMF* mycophenolate mofetil, *CSA* cyclosporine, *TAC* tacrolimus, *LEF* leflunomide, *PRED* prednisone, prednisolone or methylprednisolone, *AZA* azathioprine, *RTX* rituximab, *HD* high dose, *LD* low dose, when not specified, it indicates standard dose

Significant odds ratios are in italics

The odds ratios were transformed to relative risk (RR) and risk difference was done to allow ease for interpretation for clinicians and patients

### Treatment failure: renal relapse/renal flare

Thirteen studies with 1,108 patients provided data; 11 were two-arm and two were three-arm studies. MMF and CYC were associated with a lower rate of renal relapse/flare compared to PRED and MMF was associated with a lower rate of renal relapse/flare than AZA (Table 3). The quality of evidence was rated as moderate (downgraded for imprecision). The event rates ranged from 14 to 49 % and are shown in more detail in an additional file (see Additional file 6).

**Table 3:** Comparison of all lupus nephritis treatments for a composite of renal relapse or renal flare

Treatment	Reference	Odds ratio (95 % CrI)	Relative risk (95 % CrI)	Risk difference % (95 % Crl)
AZA	PRED	0.33 (0.08, 1.23)	0.50 (0.17, 1.12)	−0.24 (−0.47, 0.05)
MMF		*0.17 (0.04, 0.67)*	*0.29 (0.09, 0.78)*	−*0.34 (*−*0.53,* −*0.09)*
CYC		*0.18 (0.05, 0.58)*	*0.31 (0.11, 0.71)*	−*0.33 (*−*0.51,* −*0.12)*
CSA		0.24 (0.03, 1.60)	0.39 (0.07, 1.26)	−0.29 (−0.52, 0.11)
CYC + AZA		0.32 (0.05, 1.67)	0.48 (0.10, 1.29)	−0.25 (−0.50, 0.12)
MMF-AZA		0.41 (0.03, 5.46)	0.58 (0.07, 1.83)	−0.20 (−0.51, 0.36)
MMF	AZA	*0.51 (0.32, 0.87)*	*0.59 (0.38, 0.90)*	−*0.10 (*−*0.20,* −*0.02)*
CYC		0.55 (0.24, 1.26)	0.62 (0.32, 1.21)	−0.09 (−0.26 0.03)
CSA		0.74 (0.18, 2.95)	0.80 (0.23, 2.01)	−0.04 (−0.23, 0.23)
CYC + AZA		0.95 (0.27, 3.09)	0.96 (0.34, 2.11)	−0.01 (−0.19, 0.24)
MMF-AZA		1.23 (0.14, 12.15)	1.16 (0.19, 3.76)	0.04 (−0.25, 0.53)
CYC	MMF	1.06 (0.44, 2.58)	1.05 (0.51, 2.29)	0.01 (−0.13, 0.12)
CSA		1.43 (0.32, 6.17)	1.33 (0.36, 3.81)	0.04 (−0.12, 0.35)
CYC + AZA		1.84 (0.51, 6.00)	1.62 (0.56, 3.85)	0.08 (−0.07, 0.35)
MMF-AZA		2.39 (0.27, 23.30)	1.94 (0.31, 6.94)	0.13 (−0.12, 0.63)
CSA	CYC	1.35 (0.27, 6.52)	1.27 (0.31, 3.86)	0.04 (−0.14, 0.37)
CYC + AZA		1.72 (0.44, 6.37)	1.53 (0.49, 3.83)	0.08 (−0.09, 0.3)
MMF-AZA		2.24 (0.24, 23.15)	1.85 (0.28, 6.46)	0.13 (−0.13, 0.63)
CYC + AZA	CSA	1.27 (0.20, 8.09)	1.20 (0.30, 5.35)	0.03 (−0.29, 0.35)
MMF-AZA		1.67 (0.13, 25.89)	1.43 (0.19, 8.68)	0.08 (−0.31, 0.62)
MMF-AZA	CYC + AZA	1.30 (0.22, 9.13)	1.20 (0.28, 3.68)	0.04 (−0.01, 0.46)
Random-effects model	Residual deviance	32.06 vs. 28 data points		
	Deviance information criteria	146.869		
Fixed-effects model	Residual deviance	34.31 vs. 28 data points		
	Deviance information criteria	147.018		

Based on 13 RCTs with 1108 patients: 11 two-arm trials and two three-arm trials

Significant odds ratios are in italics

For absolute rates for events used for calculation of risk difference, please see Appendix 6

*OR* odds ratio, *RR* relative risk, *RD* risk difference, *CrI* credible interval, *CYC* cyclophosphamide, M*MF* mycophenolate mofetil, *CSA* cyclosporine, *TAC* tacrolimus, L*EF* leflunomide, *PRED* prednisone, prednisolone or methylprednisolone, *AZA* azathioprine, *RTX* rituximab

CYC + AZA CYC with AZA, MMF-AZA MMF followed by AZA

Merged doses for PRED and CYC and comparing only between treatments. We did not lose any study, but it is a limitation of this analysis

### Amenorrhea/ovarian failure

Eight RCTs with 839 patients provided data; seven were two-arm and one trial was a three-arm trial. CYC was more likely than MMF and PRED to be associated with amenorrhea/ovarian failure (Table 4). CYC LD was associated with higher risk of amenorrhea/ovarian failure than MMF. The quality of evidence was rated as moderate (downgraded for imprecision). Absolute event rates ranged from 8 to 61 % and are shown in more detail in an additional file (see Additional file 6).

**Table 4:** Comparison of all lupus nephritis treatments for fertility issues (amenorrhea or ovarian failure)

Treatment	Reference	OR (95 % CrI)	RR (95 % CrI)	RD % (95 % Crl)
CYC	PRED	*3.81 (1.26, 14.31)*	*2.64 (1.18, 7.51)*	*0.25 (0.04, 0.47)*
MMF		0.48 (0.09, 3.04)	0.52 (0.11, 2.58)	−0.07 (−0.23, 0.13)
AZA		2.74 (0.26, 28.27)	2.12 (0.30, 8.18)	0.17 (−0.15, 0.65)
CYC LD		9.06 (0.63, 121.50)	3.79 (0.68, 11.71)	0.45 (−0.06, 0.81)
MMF	CYC	*0.13 (0.03, 0.41)*	*0.20 (0.05, 0.56)*	−*0.31 (*−*0.47,* −*0.16)*
AZA		0.69 (0.09, 5.08)	0.79 (0.14, 1.95)	−0.08 (−0.37, 0.36)
CYC LD		2.23 (0.23, 23.20)	1.46 (0.34, 2.71)	0.19 (−0.27, 0.55)
AZA	MMF	5.25 (0.78, 54.39)	3.68 (0.81, 19.08)	0.22 (−0.02, 0.68)
CYC LD		*16.35 (1.97, 247.70)*	*6.49 (1.72, 30.95)*	*0.50 (0.06, 0.87)*
CYC LD	AZA	3.20 (0.55, 21.24)	1.69 (0.68, 6.12)	0.22 (−0.11, 0.60)
Random-effects model	Residual deviance	15.93 vs. 17 data points		
	Deviance information criteria	72.233		
Fixed-effects model	Residual deviance	16.16 vs. 17 data points		
	Deviance information criteria	72.069		

Based on eight RCTs with 839 patients: seven two-arm trials and one three-arm trial

Significant odds ratios are in italics

For absolute rates for events used for calculation of risk difference, please see Appendix 6

*OR* odds ratio, *RR* relative risk, *RD* risk difference, *CrI* credible interval, *HD* high dose, *LD* low dose; when not specified, it indicates standard dose, *CYC* cyclophosphamide, *MMF* mycophenolate mofetil, *CSA* cyclosporine, *TAC* tacrolimus, *LEF* leflunomide, *PRED* prednisone, prednisolone or methylprednisolone, *AZA* azathioprine, *RTX* rituximab

The odds ratios were transformed to relative risk (RR) and risk difference was done to allow ease for interpretation for clinicians and patients

### Bone marrow toxicity: cytopenia (including leucopenia)

Sixteen trials provided data on 2257 patients: 15 were two-arm and one was a three-arm trial. Compared to MMF, several immunosuppressive drugs were associated with a higher risk of cytopenia: CYC, AZA, CYC LD, and CYC HD (Table 5). The quality of evidence was rated as moderate (downgraded for imprecision). Absolute event rates ranged from 7 to 30 % and are shown in more detail in an additional file (see Additional file 6).

**Table 5:** Comparison of all lupus nephritis treatments for bone marrow toxicity (cytopenia including leucopenia)

Treatment	Reference	OR (95 % CrI)	RR (95 % CrI)	RD % (95 % Crl)
CYC SD	MMF	*2.16 (1.14, 4.03)*	*1.99 (1.13, 3.37)*	*0.07 (0.01, 0.16)*
AZA SD		*2.42 (1.01, 7.07)*	*2.19 (1.01, 5.06)*	*0.09 (0.00, 0.27)*
CYC LD		*5.31 (1.26, 24.65)*	*4.03 (1.23, 10.11)*	*0.22 (0.02, 0.56)*
CYC HD		*5.36 (1.95, 18.08)*	*4.06 (1.81, 8.96)*	*0.22 (0.07, 0.48)*
RTX + MMF SD		3.50 (0.61, 23.14)	2.95 (0.63, 9.43)	0.14 (−0.03, 0.56)
AZA SD	CYC SD	1.11 (0.39, 4.03)	1.09 (0.44, 3.07)	0.01 (−0.11, 0.22)
CYC LD		2.45 (0.51, 13.07)	2.01 (0.56, 5.91)	0.15 (−0.08, 0.50)
CYC HD		2.47 (0.77, 9.99)	2.03 (0.80, 5.38)	0.15 (−0.04, 0.43)
RTX + MMF SD		1.63 (0.26, 11.77)	1.49 (0.30, 5.31)	0.07 (−0.13, 0.49)
CYC LD	AZA SD	2.16 (0.42, 10.83)	1.80 (0.49, 5.39)	0.13 (−0.12, 0.47)
CYC HD		2.20 (0.56, 8.96)	1.83 (0.65, 5.16)	0.13 (−0.10, 0.40)
RTX + MMF SD		1.43 (0.18, 11.42)	1.33 (0.23, 5.33)	0.05 (−0.21, 0.48)
CYC HD	CYC LD	1.01 (0.29, 3.75)	1.01 (0.46, 2.74)	0.00 (−0.28, 0.24)
RTX + MMF SD		0.66 (0.06, 7.15)	0.74 (0.12, 3.78)	−0.07 (−0.48, 0.40)
RTX + MMF SD	CYC HD	0.65 (0.08, 5.36)	0.73 (0.13, 2.81)	−0.08 (−0.41, 0.37)
Random-effects model	Residual deviance		31.43 vs. 33 data points	
	Deviance information criteria		165.076	
Fixed-effects model	Residual deviance		38.78 vs. 33 data points	
	Deviance information criteria		167.984	

Based on 16 RCTs with 2257 patients: 15 two-arm trials and one three-arm trial

Significant odds ratios are in italics

For absolute rates for events used for calculation of risk difference, please see Appendix 6

*OR* odds ratio, *RR* relative risk, *RD* risk difference, *CrI* credible interval, *HD* high dose, *LD* low dose; when not specified, it indicates standard dose, *CYC* cyclophosphamide, *MMF* mycophenolate mofetil, *CSA* cyclosporine, *TAC* tacrolimus, *LEF* leflunomide, *PRED* prednisone, prednisolone or methylprednisolone, *AZA* azathioprine, *RTX* rituximab

MMF-AZA, MMF followed by AZA

RTX + MMF, RTX combined with MMF

Estimates for LEF HD were obtained from data from only one study and were therefore imprecise

The odds ratios were transformed to relative risk (RR) and risk difference was done to allow ease for interpretation for clinicians and patients

### Sensitivity analyses limited to partial or complete renal remission only

An additional file shows in detail the odds ratios for comparisons of immunosuppressive drugs and corticosteroids in lupus nephritis for partial/complete remission, a sensitivity analysis (renal response excluded from the composite outcome; see Additional file 7). Results were similar to the main analyses, with minor exceptions. Thus, most observations from the main analysis were confirmed in this sensitivity analysis.

### Rankograms and staircase diagrams

**Fig. 3:**
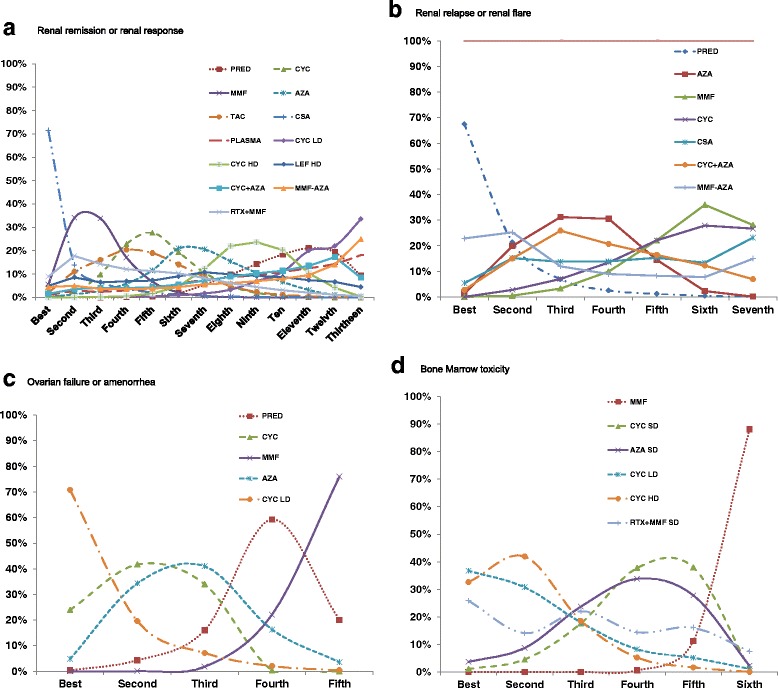
Rankograms for composite study outcomes in lupus nephritis, renal remission or renal response (**a**), renal relapse or renal flare (**b**), fertility issues (**c**), and bone marrow toxicity (**d**). Legend: This two-dimensional plot show on the *x*-axis (*horizontal*) the possible ranks of the treatment from best to the last ranks and on the *y*-axis (*vertical*) the probability of each of the treatments to assume those possible ranks for each outcome. For example, for renal relapse/flare (an undesired outcome), the highest probability was evident with corticosteroids alone, followed by MMF-AZA, followed by AZA and others

Figure 2 shows the Rankograms for various treatments for the outcomes of interest. Among the top ranked were CSA for renal remission/response, prednisone for renal relapse/flare, CYC for ovarian failure/amenorrhea and CYC for bone marrow toxicity (Fig. 3). An additional file shows in detail comparisons of various treatments to each other for each outcome, another way to visualize the key comparisons between treatments (see Additional file 8).

## Discussion

In this systematic review and NMA of outcomes in patients with lupus nephritis, we made several important observations. Results of this study are of great value to both medical and patient communities, given the growing and renewed focus on patient-centered education and outcomes. We directly compared benefits and risks of medications that are used to treat SLE nephritis. The information presented here served as the foundation for a decision-aid tool that can be easily understood that is being tested in a randomized trial, currently underway. Given the novel NMA methodology and our ability to perform indirect comparisons using the NMA, several of our findings are new and merit further discussion.

We noted differences between immunosuppressive drugs regarding renal remission/response. Interestingly, high-dose CYC was significantly less effective than MMF in leading to renal remission/response with a relative risk of 0.65 and an odds ratio of 0.40. This is an interesting finding and is consistent with the trial data from Ginzler et al. that found that MMF was superior to IV CYC in inducing complete renal remission [37].

We also found that CYC LD (which includes EURO-lupus regimen) was inferior to MMF, CSA and CYC standard dose for renal remission/response. We realize that not all trials are same; however, the superiority of these commonly used immunosuppressive drugs to CYC LD is worth some discussion. This benefit of using low-dose CYC must be weighed against the potential harms of using MMF, CSA or CYC standard dose in a patient with lupus nephritis. Several other differences we noted might be of interest to clinicians and patients when making treatment decisions for lupus nephritis.

We confirmed that immunosuppressive drugs were more effective than corticosteroids alone (most often prednisone or prednisolone) for renal remission/response by two to four times, that is, CYC, MMF, CSA, and tacrolimus led to renal remission significantly more often than corticosteroids alone. The lower risk of renal relapse or flare with MMF and CYC compared to corticosteroids alone is also supportive of this finding and not surprising. Other immunosuppressives (AZA, CSA, CYC-AZA, MMF-AZA) also seemed to be possibly more effective than corticosteroids alone for preventing renal relapse/flare, but did not reach significance.

Significant differences were found in the risk of amenorrhea/ovarian failure (fertility issues). These are important findings, since a disproportionate number of young women are affected by lupus. An increased risk of amenorrhea/ovarian failure was seen with CYC compared to MMF and PRED, which confirms a long-known clinical observation, but now provides estimates of the comparative risk. Ovarian failure is an important discussion point during lupus treatment decision-making in young women, especially when the use of CYC is considered.

We found that CYC SD, CYC LD, CYC HD, and AZA had two to five times higher risk of cytopenia than MMF. Our comprehensive NMA provided a robust treatment estimates for these comparisons that can be shared with patients in a more understandable format at the time of treatment decision-making for lupus nephritis in regular clinical care.

Our study has several limitations. Meta-analyses are observational studies and therefore can have limitations of any observational study. Heterogeneity is an issue with all meta-analyses, including NMA. We assessed for clinical heterogeneity of studies prior to conducting the analyses, with the help of two clinicians (including a rheumatologist and a lupus expert) who assessed for systematic differences between study populations and disease stage by medications regimens. We noted some clinical heterogeneity between trials, but did not find major systematic differences by the type of medication used. We acknowledge that no two clinical trials are alike. This applies to our NMA as well, and therefore, the results should be interpreted with some caution. Another study limitation may be that we searched two databases.

The NMA incorporates both direct and indirect comparisons. As can be seen from the network diagrams for these analyses, for some outcomes assessed in this study, direct comparisons were fewer, which indicates that most evidence came from indirect comparisons; addition of data from direct comparator trials in future NMA would increase the confidence in these findings. Due to multiple NMA comparisons performed, some findings may be resulting from chance. However, we think that type II error, i.e., missing important differences due to small sample size, is a bigger concern, since most included trials for this rare condition, were of small size. For comparisons with large relative effects (odds or risk ratios), one should keep in mind the underlying rates, which varied between outcomes and were very low for some composite outcomes. In these cases, the absolute difference between treatments is still small, even though relative effect may be five or ten times, or 0.05 times. Comparisons with wider confidence intervals must be interpreted with caution, since these indicate small study numbers, or a lower confidence in the certainty of the estimate. It is possible (and even likely sometimes) that addition of more data from studies in the future may change these estimates.

## Conclusions

This systematic review and NMA provides the most current, comprehensive comparison of immunosuppressive medications and corticosteroids used to treat lupus nephritis. These findings must be interpreted considering between study heterogeneity. This comparative effectiveness research provides estimates of key benefits and harms that would be of interest to both physicians and their patients. The state-of-the-art methodology used here should be duplicated for other diseases where physicians and patients will benefit from comparisons of commonly used treatments.

## Additional files

Additional file 1: Detailed definitions of efficacy outcomes and medication doses. (DOCX 22 kb) Additional file 2: A. PubMed search strategy for updating the American College of Rheumatology (ACR) lupus treatment guideline and the Cochrane library searches. B. Second Search strategy to identify any lupus trial for side effects of medications in PubMed and Scopus databases (data abstracted, but not used due to scant data and the possibility of heterogeneity in patient population, i.e., lupus nephritis vs. lupus with all other manifestations). (DOC 25 kb) Additional file 3: PRISMA NMA checklist of items to include when reporting a systematic review involving a network meta-analysis. (DOCX 97 kb) Additional file 4: Characteristics of included studies. (DOCX 41 kb) Additional file 5: All Comparisons of various treatments for the composite end-point of renal remission or renal response (includes partial remission/complete remission/renal response). (DOCX 28 kb) Additional file 6: Event rates for the outcomes of interest. (DOCX 19 kb) Additional file 7: Sensitivity analysis including partial/complete renal remission only. (DOCX 29 kb) Additional file 8: Staircase diagrams for key outcomes. (DOCX 26 kb)

## Abbreviations

NMA: Network meta-analysis
OR: Odds ratio
CrI: Credible interval
MTC: Mixed treatment comparison
AHRQ: Agency for Healthcare Research and Quality
UAB: University of Alabama at Birmingham
RCTs: Randomized controlled trials
CCTs: Controlled clinical trials
PRED: Corticosteroids, including prednisone
CYC: Cyclophosphamide
MMF: Mycophenolate mofetil
AZA: Azathioprine
CSA: Cyclosporine
TAC: Tacrolimus
RTX: Rituximab
LD: Low-dose/duration
HD: High-dose/duration
SD: Standard-dose/duration
IV: Intravenously
PO: Orally
PLASMA: Plasmapharesis
