# Supplementary material for: Comparative effectiveness of immunosuppressive drugs and corticosteroids for lupus nephritis: a systematic review and network meta-analysis
Source: Syst Rev. 2016 Sep 13;5(1):155. doi: 10.1186/s13643-016-0328-z (PMC5020478; doi:10.1186/s13643-016-0328-z)
Supplement: Additional file 1: — Detailed definitions of efficacy outcomes and medication doses. (DOCX 22 kb) [file 13643_2016_328_MOESM1_ESM.docx]

**ADDITIONAL FILES**

**Additional File 1. Detailed definitions of efficacy outcomes and medication doses**

1. End-stage renal disease (ESRD): defined as renal disease with progression to dialysis, i.e., requiring renal replacement therapy.
2. Renal response: was defined as: (1) decrease in urine protein-creatinine ratio (calculated from a 24-hour urine collection) **>**50% and **<**3 in patients **>**3 at baseline, or by **>**50% in patients **<**3 at baseline; and (2) stabilization to within 25% of baseline or improvement in serum creatinine level by 24 weeks.
3. Renal relapse**:** defined as worsening in serum creatinine or proteinuria. In one study a renal relapse was defined as: (i) the recurrence or the development of nephrotic syndrome (serum albumin ≤3.5 g/dl and 24 h proteinuria ≥3 g; this type of renal flare is further referred to as ‘nephrotic syndrome’); (ii) renal impairment (≥33% increase of serum creatinine within a 1-month period directly attributed to lupus and confirmed 1 week later; flare referred to as ‘renal impairment’); or (iii) a threefold increase of 24 h proteinuria within a 3-month period accompanied by microscopic hematuria (defined as a number of red blood cells (RBC) per high power field superior to upper normal limit for the local laboratory) and ≥33% reduction of serum C3 level within a 3-month period (this definition of renal flare was only applicable to those patients with low-grade baseline 24 h proteinuria (≥0.5 g and <1 g); this type of renal flare is further referred to as ‘proteinuria increase’).[^23^](#_ENREF_23) In another study it was defined as the doubling of serum creatinine (nephritic flare), or increase in proteinuria (proteinuric flares; unspecified increase).[^23^](#_ENREF_23)
4. Renal failure: as defined in studies, which was bit variable, but commonly defined as significant renal insufficiency such as doubling of creatinine or decrease in glomerular filtration rate (GFR) >20%, worsening of proteinuria, or occurence of chronic renal failure etc.
