# Supplementary material for: Comparative effectiveness of immunosuppressive drugs and corticosteroids for lupus nephritis: a systematic review and network meta-analysis
Source: Syst Rev. 2016 Sep 13;5(1):155. doi: 10.1186/s13643-016-0328-z (PMC5020478; doi:10.1186/s13643-016-0328-z)
Supplement: Additional file 2: — A. PubMed search strategy for updating the American College of Rheumatology (ACR) lupus treatment guideline and the Cochrane library searches. B. Second Search strategy to identify any lupus trial for side effects of medications in PubMed and Scopus databases (data abstracted, but not used due to scant data and the possibility of heterogeneity in patient population, i.e., lupus nephritis vs. lupus with all other manifestations). (DOC 25 kb) [file 13643_2016_328_MOESM2_ESM.doc]

**Additional File 2.**

**2A.** PubMed search strategy for updating the American College of Rheumatology (ACR) lupus treatment guideline and the Cochrane library searches

(Lupus[text word] OR "Lupus Vulgaris"[MeSH] OR "Lupus Erythematosus, Cutaneous"[MeSH] OR "Lupus Erythematosus, Systemic"[Mesh]) **AND** ("Kidney Diseases"[MeSH] OR nephropath*[text word] OR Transplants[MeSH] OR Transplantation[MesH] OR transplantation[subheading] OR transplant*[text word] OR "Kidney"[Mesh] OR Kidney*[text word] OR Renal*[text word] OR "End Stage Renal Disease"[text word] OR ESRD[text word] OR Glomerulonephr*[text word] OR "GN"[text word] OR "crescentic GN"[text word]) **NOT** ("animals"[MeSH] NOT "humans"[MeSH])

**2B. Second Search strategy to identify any lupus trial for side effects of medications in PubMed and Scopus databases (data abstracted, but not used due to scant data and the possibility of heterogeneity in patient population, i.e., lupus nephritis vs. lupus with all other manifestations)**

1. (Lupus Erythematosus, Systemic [mh:noexp] OR “systemic lupus erythematosus” [tw]) AND (Randomized Controlled Trial [pt] OR Randomized Controlled Trials as Topic [mh] OR “randomized controlled” [tw] OR “randomised controlled” [tw]) AND Adult [mh] AND English [la] NOT Nephritis OR Glomerulonephritis

2. (Lupus Erythematosus, Systemic [mh:noexp] OR “systemic lupus erythematosus” [tw]) AND (Randomized Controlled Trial [pt] OR Randomized Controlled Trials as Topic [mh] OR “randomized controlled” [tw] OR “randomised controlled” [tw]) AND English [la] NOT Child OR adolescent OR infant OR newborn OR preschool NOT Nephritis OR Glomerulonephritis
