# Supplementary material for: Comparative effectiveness of immunosuppressive drugs and corticosteroids for lupus nephritis: a systematic review and network meta-analysis
Source: Syst Rev. 2016 Sep 13;5(1):155. doi: 10.1186/s13643-016-0328-z (PMC5020478; doi:10.1186/s13643-016-0328-z)
Supplement: Additional file 4: — Characteristics of included studies. (DOCX 41 kb) [file 13643_2016_328_MOESM4_ESM.docx]

**Additional File 4.** Characteristics of included studies

|  | **Author** | **Year** | **Title** | **Induction/ Maintenance** | **Country** | **Setting** | **Study Design** | **N** | **Treatment group: Male vs. Female or % female or Total N** |
| --- | --- | --- | --- | --- | --- | --- | --- | --- | --- |
| 1 | Austin | 2009 | Randomized, controlled trial of prednisone, cyclophosphamide, and cyclosporine in lupus membranous nephropathy. J Am Soc Nephrol 2009;20:901–11. | Induction | USA | NIH | RCT | 42 | PRED: M/F: 3/12 IV CYC: M/F: 3/12 CSA: M/F: 1/11 |
| 2 | Carette | 1983 | Controlled studies of oral immunosuppressive drugs in lupus nephritis. A long-term follow-up. Ann Intern Med. 1983;99(1):1-8. [148] | Induction and maintenance | USA | NIH | RCT | 53 | PRED: M/F: 3/12 AZA: M/F: 2/18 CYC: M/F: 5/13 |
| 3 | Steinberg | 1991 | Long-term preservation of renal function in patients with lupus nephritis receiving treatment that includes cyclophosphamide versus those treated with prednisone only. Arthritis Rheum 1991;34:945–50. | Follow-up | USA | NIH | RCT | 111 | PRED, N=30; AZA, N=20; PO CYC, N=18; IV CYC, N=20; AZA +CYC, N=23 |
| 4 | Donadio | 1976 | Progressive lupus glomerulonephritis. Treatment with prednisone and combined prednisone and cyclophosphamide. Mayo Clin Proc. 1976;51(8):484-94. [154] | Induction | USA | Single center | RCT | 39 | PRED, N=20;  CYC + PRED, N=19 |
| 5 | Pohl | 1991 | Plasmapheresis does not increase the risk for infection in immunosuppressed patients with severe lupus nephritis. The Lupus Nephritis Collaborative Study Group. Ann Intern Med. 1991;114(11):924-9 [157] | Induction, follow-up | USA | Multicenter | RCT | 86 | Standard group, 84.8% F; plasmapharesis group, 82.5% F |
| 6 | Mok | 2001 | Treatment of diffuse proliferative lupus glomerulonephritis: a comparison of two cyclophosphamide-containing regimens. Am J Kidney Dis 2001;38:256–64. | Induction and maintenance | Hong Kong | 2 hospital sites | RCT | 43 | IV pulse CYC, M/F: 1/21;  Pulse CYC followed by AZA, M/F: 1/20 |
| 7 | Hu | 2002 | Mycophenolate mofetil vs cyclophosphamide therapy for patients with diffuse proliferative lupus nephritis. Chin Med J (Engl). 2002;115(5):705-9. [162] | Induction | China | Single center | RCT | 46 | MMF group = 19F; CYC group = 19 F |
| 8 | Wang | 2007 | Induction therapies for class IV lupus nephritis with non-inflammatory necrotizing vasculopathy: mycophenolate mofetil or intravenous cyclophosphamide. Lupus. 2007;16(9):707-12. [165] | Induction | China | Single center | RCT | 20 | MMF group, N= 9; CYC group, N = 11 |
| 9 | Isenberg | 2010 | Influence of race/ethnicity on response to lupus nephritis treatment: the ALMS study. Rheumatology (Oxford) 2010;49:128–40. | Induction | Multinational | Multicenter | RCT | 370 | Induction therapy M/F: 57/313; Maintenance therapy M/F: 32/195 |
| 10 | Radhakrishnan | 2010 | Mycophenolate mofetil and intravenous cyclophosphamide are similar as induction therapy for class V lupus nephritis. Kidney Int 2010;77:152–60. | Induction pooled data | USA/multinational | Multicenter; two RCTs | Pooled analysis of 2 large RCTs | 84 | US study: M/F: 4/20  ALMS study subgroup: M/F: 17/43 |
| 11 | Wang | 2008 | Induction treatment of proliferative lupus nephritis with leflunomide combined with prednisone: a prospective multi-centre observational study. Lupus. 2008;17(7):638-44. [173] | Induction | China | Multicenter | RCT | 110 | LEF, M/F: 10/60;  CYC, M/F: 3/37 |
| 12 | Appel [ALMS study] | 2009 | Appel GB, Contreras G, Dooley MA, Ginzler EM, Isenberg D, Jayne D, et al. Mycophenolate mofetil versus cyclophosphamide for induction treatment of lupus nephritis. Journal of the American Society of Nephrology 2009;20(5):1103–12. | Induction and Maintenance | Multinational | NIH trial, Multicenter | RCT, open-label, parallel-group | 370 | Induction therapy M/F: 57/313; Maintenance therapy M/F: 32/195 |
| 13 | Austin | 1986 | Austin HA, Klippel JH, Balow JE, le Riche NG, Steinberg AD, Plotz PH, et al. Therapy of lupus nephritis. Controlled trial of prednisone and cytotoxic drugs. New England Journal of Medicine 1986;314(10):614–9. | Induction | USA | NIH trials, Multicenter | RCT, open | 107 | M/F: 15/92 |
| 14 | Balletta | 1992 | Balletta M, Sabella D, Magri P, Sepe V, Stanziale P, Di Luccio R, et al. Cyclosporin plus steroids versus steroids alone in the treatment of lupus nephritis. Contributions to Nephrology 1992;99:129–30. | Induction | Italy | NS | RCT | 10 | PRED: M/F: 1/4  PRED+CSA: M/F: 0/5 |
| 15 | Bao | 2008 | Bao H, Liu ZH, Xie HL, Hu WX, Zhang HT, Li LS. Successful treatment of class V+IV lupus nephritis with multi-target therapy. Journal of the American Society of Nephrology 2008;19(10):2001–10. | Induction | China | Single center | RCT, open-label | 40 | MMF+TAC+PRED: M/F: 4/16 IV CYC: M/F: 2/18 |
| 16 | Barron | 1982 | Barron KS, Person DA, Brewer EJ Jr, Beale MG, Robson AM. Pulse methylprednisolone therapy in diffuse proliferative lupus nephritis. Journal of Pediatrics 1982;101 (1):137–41. | Induction | USA | Single center | Quasi-RCT | 22 | Oral PRED HD: M/F: 2/13 IV pulse PRED, then PO PRED: M/F: 1/6 |
| 17 | Boumpas | 1992 | Boumpas DT, Austin HA, Vaughn EM, Klippel JH, Steinberg AD, Yarboro CH, et al. Controlled trial of pulse methylprednisolone versus two regimens of pulse cyclophosphamide in severe lupus nephritis. Lancet 1992; 340(8822):741–5. | Induction | USA | NS | RCT | 65 | Pulse PRED: M/F: 1/24 Pulse CYC: M/F: 3/17 Pulse CYC, then quarterly: M/F: 1/19 |
| 18 | Cade | 1973 | Cade R, Spooner G, Schlein E, Pickering M, DeQuesada A, Holcomb A, et al. Comparison of azathioprine, prednisone, and heparin alone or combined in treating lupus nephritis. Nephron 1973;10(1):37–56. | Induction | USA | Teaching hospital | Quasi-RCT | 54 | PRED HD: M/F: 3/12 AZA: M/F: 1/12 AZA+PRED: M/F: 3/10 AZA+ heparin: M/F: 6/7 |
| 19 | Chan | 2000 | Chan TM, Li FK, Tang CS,Wong RW, Fang GX, Ji YL, et al. Efficacy of mycophenolate mofetil in patients with diffuse proliferative lupus nephritis. Hong Kong-Guangzhou Nephrology Study Group. New England Journal of Medicine 2000;343(16):1156–62. | Induction and maintenance | Hong Kong | Multicenter | RCT | 42 | PRED +MMF: M/F: 6/26 PRED+CYC, then PRED+AZA: M/F: 4/26 |
| 20 | Chen | 2011 | Chen W, Tang X, Liu Q, Chen W, Fu P, Liu F, et al. Shortterm outcomes of induction therapy with tacrolimus versus cyclophosphamide for active lupus nephritis: A multicenter randomized clinical trial. American Journal of Kidney Diseases 2011;57(2):235–44. | Induction | China | Multicenter | RCT | 81 | PRED +TAC: M/F: 5/37 PRED +CYC: M/F: 7/32 |
| 21 | Clark | 1981 | Clark WF, Lindsay RM, Cattran DC, Chodirker WB, Barnes CC, Linton AL. Monthly plasmapheresis for systemic lupus erythematosus with diffuse proliferative glomerulonephritis: a pilot study. CMAJ Canadian Medical Association Journal 1981;125(2):171–4. | Induction | Canada | Outpatient | RCT | 12 | Conventional therapy: N=6  Conventional therapy +Plasmapharesis: N=6 |
| 22 | Clark | 1984 | Clark WF,Williams W, Cattran DC, Balfe JW, Chodirker WB, Koval JJ, et al. A controlled trial of chronic plasma exchange therapy in S.L.E. nephritis [abstract]. Kidney International 1984;25(1):161 | Induction | Canada and West Indies | Multicenter | RCT | 39 | Conventional therapy: M/F: 1/19 Conventional therapy +Plasmapharesis: M/F: 5/15 |
| 23 | Contreras | 2002 | Contreras G, Pardo V, Leclercq B, Lenz O, Tozman E,  O’Nan P, et al. Sequential therapies for proliferative lupus  nephritis. New England Journal of Medicine 2004;350(10):  971–80. [MEDLINE: 14999109] &  Contreras G, Pardo V, Leclercq B, Gomez E, Reich J, O’Nan P, et al. Maintenance therapy for proliferative forms of lupus nephritis: a randomized clinical trial comparing quarterly intravenous cyclophosphamide (IVCY) versus oral mycophenolate mofetil (MMF) or azathioprine (AZA) [abstract]. Journal of the American Society of Nephrology 2002;13(Program & Abstracts):15A | Maintenance | USA | Single center | RCT, Open-label | 59 | IV CYC: M/F: 1/19 AZA: M/F: 2/18 MMF: M/F: 1/19 |
| 24 | Cyclofa-Lune Study | 2010 | Zavada J, Pesickova S, Rysava R, Olejarova M,Horak P, Hmcir Z, et al. Cyclosporine A or intravenous cyclophosphamide for lupus nephritis: the Cyclofa-Lune study. Lupus 2010;19(11):1281–9. [ | Induction and maintenance | European countries | Multicenter | RCT, open label | 40 | CYC: M/F: 6/15 CSA: M/F: 5/14 |
| 25 | Derksen | 1988 | Derksen RH, Hene RJ, Kallenberg CG, Valentijn RM, Kater L. Prospective multicentre trial on the short- term effects of plasma exchange versus cytotoxic drugs in corticosteroid-resistant lupus nephritis. Netherlands Journal of Medicine 1988;33(3-4):168–77. | Induction | The Netherlands | Multicenter | RCT | 20 | AZA or CYC: M/F 3/8 Plasmapharesis: M/F: 2/7 |
| 26 | Donadio | 1974 | Donadio JVJ, Holley KE, Wagoner RD, Ferguson RH, McDuffie FC. Further observations on the treatment of lupus nephritis with prednisone and combined prednisone and azathioprine. Arthritis & Rheumatism 1974;17(5): 573–81. | Induction | USA | NS | RCT | 16 | M/F: 2/14  PRED vs. PRED +AZA |
| 27 | Donadio | 1978 | Donadio JV, Holley KE, Ferguson RH, Ilstrup DM. Treatment of diffuse proliferative lupus nephritis with prednisone and combined prednisone and cyclophosphamide. New England Journal of Medicine 1978; 299(21):1151–5. | Induction | USA | Single center | RCT, Open-label | 26 | PRED: M/F: 4/22 PRED +CYC: M/F: 5/19 |
| 28 | Doria | 1994 | Doria A, Piccoli A, Vesco P, Vaccaro E, Marson P, De Silvestro G, et al. Therapy of lupus nephritis. A two-year prospective study. Annales de Medecine Interne 1994;145 (5):307–11. | Induction | Italy | Single center | RCT | 18 | M/F: 2/16  Std therapy vs.  Std therapy +plasmapharesis vs. Std therapy +PRED |
| 29 | Dyadyk | 2001 | Dyadyk A, Vasilenko I, Bagriy A, Dyadyk O, Yarovaya N, Roschin Y, et al. Azathioprine and cyclophosphamide in treatment of patients with diffuse proliferative lupus nephritis - a randomized controlled study [abstract]. Nephrology Dialysis Transplantation 2001;16(6):A57. | Induction | Ukraine | NS | RCT | 59 | M/F: 9/50;  PO CYC: M/F: 4/17 PO AZA: M/F: 5/33 |
| 30 | El-Shafey | 2010 | El Shafey EM, Abdou SH, ShareefMM. Is mofetil superior to pulse intravenous cyclophosphamide for induction therapy of proliferative lupus nephritis in Egyptian patients. Clinical & Experimental Nephrology 2010;14(3):214–21. | Induction | Egypt | Single center | RCT, open-label | 47 | MMF: M/F: 1/23 Pulse IV CYC: M/F: 1/22 |
| 31 | Fu | 1998 | Fu LW, Yang LY, Chen WP, Lin CY. Clinical efficacy of cyclosporin A neoral in the treatment of paediatric lupus nephritis with heavy proteinuria. British Journal of Rheumatology 1998;37(2):217–21. | Maintenance | Taiwan | Single Center | RCT | 40 | CYC: N=20  PRED +CSA: N=20 |
| 32 | Ginzler | 2005 | Ginzler EM, Dooley MA, Aranow C, Kim MY, Buyon J, Merrill JT, et al. Mycophenolate mofetil or intravenous cyclophosphamide for lupus nephritis. New England Journal of Medicine 2005;353(21):2219–28. | Induction | USA | Single Center | RCT, open-label, non-inferiority | 140 | MMF: M/F: 10/61 CYC: M/F: 4/65 |
| 33 | Gourley | 1996 | Gourley MF, Austin HA, Scott D, Yarboro CH, Vaughan EM, Muir J, et al. Methylprednisolone and cyclophosphamide, alone or in combination, in patients with lupus nephritis. A randomized, controlled trial. Annals of Internal Medicine 1996;125(7):549–57. | Induction | USA | Single Center | RCT | 82 | PRED: M/F: 5/22 CYC: M/F: 6/21 CYC +PRED: M/F: 3/25 |
| 34 | Grootscholten [Dutch Lupus Study] | 2006 | Grootscholten C, Ligtenberg G, Hagen EC, van den Wall Bake AW, de Glas-Vos JW, Bijl M, et al. Azathioprine/methylprednisolone versus cyclophosphamide inproliferative lupus nephritis. A randomized controlled trial. Kidney International 2006;70(4):732–42 &  Grootscholten C, Bajema IM, Florquin S, et al.; Treatment with cyclophosphamide delays the progression of chronic lesions more effectively than does treatment with azathioprine plus methylprednisolone in patients with proliferative lupus nephritis.  Arthritis Rheum 2007;56:924–37. | Induction and maintenance | Netherlands | Multicenter | RCT | 87 | CYC +PRED: M/F: 6/44 AZA +PRED: M/F: 9/28 |
| 35 | Hahn | 1975 | Hahn BH, Kantor OS, Osterland CK. Azathioprine plus prednisone compared with prednisone alone in the treatment of systemic lupus erythematosus. Report of a prospective controlled trial in 24 patients. Annals of Internal Medicine 1975;83(5):597–605. | Induction | USA | Single Center | RCT | 20 | PRED: M/F: 2/11 AZA: M/F: 2/9 |
| 36 | Hong | 2007 | Hong R, Haijin Y, Xianglin W, Cuilan H, Nan C. A preliminary study of tacrolimus versus cyclophosphamide in patients with diffuse proliferative lupus nephritis [abstract]. Nephrology Dialysis Transplantation 2007;22(Suppl 6): vi276. | Induction | China | NS | RCT | 25 | Not available |
| 37 | Houssiau (Euro-Lupus Nephritis Trial) | 2002 | Houssiau FA, Vasconcelos C, D’Cruz D, Sebastiani GD, Garrido Ed Ede R, Danieli MG, et al. Immunosuppressive therapy in lupus nephritis: the Euro-Lupus Nephritis Trial, a randomized trial of low-dose versus high-dose intravenous cyclophosphamide. Arthritis & Rheumatism 2002;46(8): 2121–31. | Induction and maintenance | European | Multicenter | RCT | 90 | High-dose IV CYC followed by AZA: M/F: 3/43 Low-dose IV CYC followed by AZA M/F: 3/41 |
| 38 | Lewis | 1992 | Lewis EJ, Hunsicker LG, Lan SP, Rohde RD, Lachin JM, Lupus NCSG. A controlled trial of plasmapheresis therapy in severe lupus nephritis. The Lupus Nephritis Collaborative Study Group. New England Journal of Medicine 1992;326(21):1373–9. | Induction | USA | Multicenter | RCT | 86 | CYC +PRED: M/F: 7/33  CYC +PRED +plasmapharesis: M/F: 7/39 |
| 39 | Li | 2009a | Li EK, Tam LS, Zhu TY, Li M, Kwok CL, Li TK, et al.Is combination rituximab with cyclophosphamide better than rituximab alone in the treatment of lupus nephritis. Rheumatology 2009;48(8):892–8. | Induction | Hong Kong | Single Center | RCT | 19 | RTX: M/F; 9/9  RTX +IV CYC : M/F: 1/9 |
| 40 | Li | 2009b | Li X, Ren H, Zhang W, Xu Y, Shen P, Zhang Q, et al. Induction therapies for proliferative lupus nephritis: mycophenolate mofetil, tacrolimus and intravenous cyclophosphamide [abstract]. Journal of the American Society of Nephrology 2009;20:391A. | Induction | China | NS | RCT, Open-label | 60 | MMF: M/F: 3/17  TAC: M/F: 3/17  CYC: M/F: 2/18 |
| 41 | Lui | 1997 | Lui SF, Cheng IKP, Tong KL, Li CS, Wong KC, Chang DT, Sang WK, Chau KF. Treatment of type iv lupus nephritis (LN) - comparison of 2 triple therapy regimens: cyclosporin a (CSA), prednisolone (PRED), azathioprine (AZA) vs. oral cyclophosphamide (POCP), prednisolone, azathioprine [abstract]. Nephrology 1997;3(Suppl 1):S476 | Induction | Hong Kong | NS | RCT | 34 | Not available  CSA +PRED +AZA vs.  PO CYC + PRED +AZA |
| 42 | LUNAR Study | 2012 | Rovin BH, Furie R, Latinis K, Looney RJ, Fervenza FC, Sanchez-Guerrero J, et al. Efficacy and safety of rituximab in patients with active proliferative lupus nephritis: the Lupus Nephritis Assessment with Rituximab study. Arthritis & Rheumatism. 2012;64(4):1215-26 &  Rovin BH, Appel G, Furie R, Looney J, Latinis K, Fervenza FC, et al. Efficacy and safety of rituximab (RTX) in subjects with proliferative lupus nephritis (LN): results from the randomized, double-blind phase III LUNAR study [abstract]. Journal of the American Society of Nephrology 2009;20:77A. | Induction | Multinational | NIH trials, multicenter | RCT | 144 | Std therapy +Placebo: M/F: 5/67  Std therapy +RTX: M/F: 9/63 |
| 43 | MAINTAIN Nephritis Study | 2010 | Houssiau FA, D’CruzD, Sangle S, Remy P, Vasconcelos C, Petrovic R, et al. Azathioprine versus mycophenolate mofetil for long-term immunosuppression in lupus nephritis: results from the MAINTAIN Nephritis Trial. Annals of the Rheumatic Diseases 2010;69(12):2083–9. [MEDLINE: 20833738] | Maintenance | European | Multicenter | RCT | 105 | AZA: M/F: 4/48  MMF: M/F: 5/48 |
| 44 | Mitwalli | 2011 | Mitwalli AH, Al Wakeel JS, Hurraib S, Aisha A, Al Suwaida A, Alam A, et al. Comparison of high and low dose of cyclophosphamide in lupus nephritis patients: a long-term randomized controlled trial. Saudi Journal of Kidney Diseases & Transplantation 2011;22(5):935–40. | Induction and maintenance | Saudi Arabia | Single Center | RCT | 117 | CYC HD: M/F: 12/61 CYC LD: M/F: 5/39 |
| 45 | Mok | 2009 | Mok CC, Ying SK, Tong KH, Siu YP, To CH, Yim CW, et al. Mycophenolate mofetil versus tacrolimus for active lupus nephritis: an extended observation of a randomized controlled trial [abstract]. Annals of the Rheumatic Diseases 2009;68(Suppl 3):246. | Induction | Hong Kong, China | NS | RCT | 109 | M/F: 11/98  MMF vs. TAC |
| 46 | Moroni | 2004 | Moroni G, Doria A, Mosca M, Ferraccioli G, Todesco S, Manno C, et al. A randomized trial comparing cyclosporine versus azathioprine for maintenance therapy in diffuse lupus nephritis [abstract]. Journal of the American Society of Nephrology 2004;15(Oct):121A. | Maintenance | Italy | Multicenter | RCT | 69 | CSA: M/F: 3/33 AZA: M/F: 4/29 |
| 47 | Mulic-Bacic | 2008 | Mulic-Bacic S, Antic D, Krizic M, Hajdarovic A, Mulic E. Mycophenolate mofetil or intravenous cyclophosphamide in treatment of lupus nephritis [abstract]. Annals of the Rheumatic Diseases 2008;67(Suppl II):349. | Induction | Bosnia Herzegovina | NS | RCT | 45 | MMF, N=20  CYC, N=25 |
| 48 | My-Lupus Study | 2010 | Jayne DR, Zeher M. Enteric-coated mycophenolate sodium (ED-MPS) for the treatment of lupus nephritis - MyLupus study [abstract]. Journal of the American Society of Nephrology 2010;21:626A. | Induction | Multinational | Multicenter | RCT, open label | 81 | Not available |
| 49 | Ong | 2005 | Ong LM, Hooi LS, LimTO, Goh BL, AhmadG, Ghazalli R, et al. Randomized controlled trial of pulse intravenous cyclophosphamide versus mycophenolate mofetil in the induction therapy of proliferative lupus nephritis. Nephrology 2005;10(5):504–10. | Induction | Malaysia | Multicenter | RCT, open-label | 54 | MMF: M/F: 3/23 IV CYC: M/F: 4/15 |
| 50 | Sabry | 2009 | Sabry A, Sheashaa H, Mahmoud K, Elhuusieni A, El Dahshan K. A comparative study of two intensified pulse cyclophosphamide remission-inducing regimen for diffuse proliferative lupus nephritis: an Egyptian experience [abstract]. Nephrology Dialysis Transplantation 2007;22 (Suppl 6):vi28. | Induction | Egypt | Single Center | Quasi-RCT | 46 | CYC, HD: M/F: 4/22 CYC, LD: M/F: 2/18 |
| 51 | Sesso | 1994 | Sesso R, Monteiro M, Sato E, Kirsztajn G, Silva L, Ajzen H. A controlled trial of pulse cyclophosphamide versus pulse methylprednisolone in severe lupus nephritis. Lupus 1994;3(2):107–12. | Induction | Brazil | Single Center | RCT | 29 | IV CYC: M/F: 2/12 IC PRED: M/F: 2/13 |
| 52 | Steinberg | 1971 | Steinberg AD, Kaltreider HB, Staples PJ, Goetzl EJ, Talal N, Decker JL. Cyclophosphamide in lupus nephritis: a controlled trial. Annals of Internal Medicine 1971;75(2): 165–71. | Induction | USA | Single Center | RCT | 15 | IV CYC +PRED: M/F: 0/7 Placebo +PRED: M/F: 0/6 |
| 53 | Sundel | 2008 | Sundel RP, Lisk L. Mycophenolate mofetil compared with intravenous cyclophosphamide as induction treatment for pediatric lupus nephritis: a randomized trial [abstract 1247]. American College of Rheumatology Annual Scientific Meeting, Oct 24-29, 2008, San Francisco (CL). 2008. | Induction | Multinational | Multicenter | RCT | 24 | M/F: 5/19  MMF vs. IV CYC |
| 54 | Wallace | 1998 | Wallace DJ, Goldfinger D, Pepkowitz SH, Fichman M, Metzger Al, Schroeder JO, et al. Randomized controlled trial of pulse/synchronization cyclophosphamide/apheresis for proliferative lupus nephritis. Journal of Clinical Apheresis 1998;13(4):163–6. | Induction | Multinational | Multicenter | RCT | 19 | Std therapy: M/F: 1/8 Std therapy +Plasmapharesis: M/F: 0/9 |
| 55 | Yee | 2004 | Griffiths B, et al. EULAR randomised controlled trial of pulse cyclophosphamide and methylprednisolone versus continuous cyclophosphamide and prednisolone followed by azathioprine and prednisolone in lupus nephritis. Annals of the Rheumatic Diseases 2004;63(5):525–9. | Induction and maintenance | European | Multicenter | RCT, open label | 32 | Pulse CYC: M/F: 2/11  Continuous PO CYC followed by PO AZA: M/F: 2/14 |
| 56 | Li | 2012 | Mycophenolate mofetil or tacrolimus compared with intravenous cyclophosphamide in the induction treatment for active lupus nephritis. Nephrology Dialysis Transplantation. 2012;27(4):1467-1472. | Induction | China | Single center | RCT | 60 | MMF: M/F: 3/17 Tacrolimus: M/F: 3/17 IV CYC: M/F: 2/18 |
| 57 | Yap | 2012 | Pilot 24-month study to compare mycophenolate mofetil and tacrolimus in the treatment of membranous lupus nephritis with nephrotic syndrome. Nephrology. 2012;17(4):352-357. | Induction | Hong Kong, China | Multicenter | RCT | 16 | PRED +MMF: M/F: 2/5 PRED +TAC: M/F: 4/5 |
| 58 | Stoenoiu | 2012 | Repeat kidney biopsies fail to detect differences between azathioprine and mycophenolate mofetil maintenance therapy for lupus nephritis: data from the MAINTAIN Nephritis Trial. Nephrology Dialysis Transplantation. 2012;27(5):1924-1930. | Maintenance | Europe | Multicenter | RCT | 30 | M:F: 1/29 |
| 59 | Chen | 2012 | Outcomes of maintenance therapy with tacrolimus versus azathioprine for active lupus nephritis: a multicenter randomized clinical trial. Lupus. 2012;21(9):944-952. | Maintenance | China | Multicenter | RCT | 70 | TAC: M/F: 2/5 5/29 AZA group: M/F: 2/5 4/32 |
| 60 | Arends | 2012 | Long-term follow-up of a randomised controlled trial of azathioprine/methylprednisolone versus cyclophosphamide in patients with proliferative lupus nephritis. Annals of the Rheumatic Diseases. 2012;71(6):966-973. | Induction | Netherlands | Multicenter | RCT | 87 | CYC +PRED: M/F: 6/44 AZA +PRED: M/F: 9/28 |
| 61 | Sundel | 2012 | Efficacy of mycophenolate mofetil in adolescent patients with lupus nephritis: evidence from a two-phase, prospective randomized trial. Lupus. 2012;21(13):1433-1443. | Induction - | Multinational | Multicenter | RCT | 370 | Induction - Adults MMF: M/F: 25/150 AZA: M/F: 27/144 Maintenance - Adults MMF: M/F: 15/93  AZA: M/F: 14/89 |
| 62 | Walsh | 2013 | Mycophenolate Mofetil or Intravenous Cyclophosphamide for Lupus Nephritis With Poor Kidney Function: A Subgroup Analysis of the Aspreva Lupus Management Study. American Journal of Kidney Diseases. 2013;61(5):710-715. | Induction | Multinational | Multicenter | RCT; post-hoc analysis | 32 | CYC: M/F: 2/10  MMF: M/F: 2/18 |
| 63 | PetrI | 2010 | High-dose cyclophosphamide versus monthly intravenous cyclophosphamide for systemic lupus erythematosus: a prospective randomized trial. Arthritis Rheum. 2010;62(5):1487-1493. | Induction and maintenance | USA | 2 hospital sites | RCT | 51 | SD CYC: M/F: 3/23  HD CYC: M/F: 2/19 |
| 64 | Zeher | 2011 | Efficacy and safety of enteric-coated mycophenolate sodium in combination with two glucocorticoid regimens for the treatment of active lupus nephritis. Lupus. 2011;20(14):1484-1493. | Induction and maintenance | Multinational | Multicenter | RCT | 81 | SD PRED: M/F: 37/42 LD PRED: M/F: 29/39 |
| 65 | Dooley | 2011 | Mycophenolate versus azathioprine as maintenance therapy for lupus nephritis. N Engl J Med. 2011;365(20):1886-1895. | Maintenance | United Kingdom | Single center | RCT | 227 | MMF, M/F: 99/116; AZA, M/F: 96/111 |

NS, not specified; RCT, randomized controlled trial; Std, standard; IV, intravenous; PO, oral; NIH, National Institutes of Health

M, male; F, female

CYC, cyclophosphamide; MMF, mycophenolate mofetil; AZA, azathioprine; TAC, tacrolimus; CSA, cyclosporine; PRED, corticosteroids; LEF, leflunomide; PLASMA, plasmapharesis

HD, high dose; LD, low dose; SD, standard dose; when dose is not specified, standard dose should be inferred.
