# Supplementary material for: Comparative effectiveness of immunosuppressive drugs and corticosteroids for lupus nephritis: a systematic review and network meta-analysis
Source: Syst Rev. 2016 Sep 13;5(1):155. doi: 10.1186/s13643-016-0328-z (PMC5020478; doi:10.1186/s13643-016-0328-z)
Supplement: Additional file 5: — All Comparisons of various treatments for the composite end-point of renal remission or renal response (includes partial remission/complete remission/renal response). (DOCX 28 kb) [file 13643_2016_328_MOESM5_ESM.docx]

**Additional File 5. All Comparisons of various treatments for the composite end-point of renal remission or renal response (includes partial remission/complete remission/renal response)**

| **Treatment** | **Reference** | **OR (95% CrI)** | **RR (95% CrI)** | **RD % (95% Crl)** |
| --- | --- | --- | --- | --- |
| CYC | PRED | **2.35(1.28,4.23)** | **1.60(1.60,2.24)** | **0.21(0.06,0.34)** |
| MMF |  | **3.26(1.57,6.72)** | **1.82(1.82,2.60)** | **0.28(0.11,0.44)** |
| AZA |  | 1.80(0.80,3.87) | 1.41(1.41,2.13) | 0.14(-0.05,0.32) |
| TAC |  | **2.51(1.11,5.76)** | **1.64(1.64,2.44)** | **0.22(0.03,0.41)** |
| CSA |  | **5.69(2.02,17.61)** | **2.15(2.15,3.18)** | **0.40(0.17,0.58)** |
| PLASMA |  | 1.07(0.34,3.50) | 1.05(1.05,1.93) | 0.02(-0.20,0.30) |
| CYC LD |  | 0.75(0.21,2.39) | 0.82(0.82,1.66) | -0.06(-0.27,0.21) |
| CYC HD |  | 1.31(0.50,3.07) | 1.18(1.18,1.90) | 0.06(-0.14,0.27) |
| LEF HD |  | 1.70(0.37,7.78) | 1.37(1.37,2.54) | 0.13(-0.19,0.46) |
| CYC+AZA |  | 1.16(0.21,6.13) | 1.10(1.10,2.35) | 0.03(-0.26,0.42) |
| MMF-AZA |  | 1.03(0.12,8.60) | 1.02(1.02,2.52) | 0.01(-0.30,0.47) |
| RTX+MMF |  | 2.61(0.77,9.08) | 1.67(1.67,2.68) | 0.23(-0.06,0.49) |
| MMF | CYC | 1.38(0.91,2.21) | 1.14(1.14,1.37) | 0.08(-0.02,0.18) |
| AZA |  | 0.76(0.41,1.42) | 0.88(0.88,1.17) | -0.07(-0.22,0.08) |
| TAC |  | 1.07(0.59,2.01) | 1.03(1.03,1.32) | 0.02(-0.13,0.16) |
| CSA |  | 2.44(0.96,6.63) | 1.35(1.35,1.78) | 0.19(-0.01,0.36) |
| PLASMA |  | 0.46(0.12,1.71) | 0.66(0.66,1.26) | -0.19(-0.44,0.13) |
| CYC LD |  | **0.32(0.10,0.89)** | **0.51(0.51,0.95)** | **-0.26(-0.45,-0.03)** |
| CYC HD |  | 0.56(0.26,1.09) | 0.74(0.74,1.04) | -0.14(-0.31,0.02) |
| LEF HD |  | 0.73(0.17,2.96) | 0.86(0.86,1.44) | -0.08(-0.37,0.23) |
| CYC+AZA |  | 0.50(0.10,2.40) | 0.69(0.69,1.36) | -0.17(-0.45,0.19) |
| MMF-AZA |  | 0.44(0.05,3.41) | 0.64(0.64,1.47) | -0.20(-0.50,0.25) |
| RTX+MMF |  | 1.11(0.38,3.40) | 1.05(1.05,1.51) | 0.03(-0.23,0.26) |
| AZA | MMF | 0.55(0.28,1.06) | 0.77(0.77,1.02) | -0.14(-0.30,0.01) |
| TAC |  | 0.77(0.42,1.45) | 0.90(0.90,1.15) | -0.06(-0.21,0.08) |
| CSA |  | 1.76(0.63,5.20) | 1.18(1.18,1.61) | 0.12(-0.11,0.31) |
| PLASMA |  | 0.33(0.08,1.30) | 0.58(0.58,1.11) | -0.26(-0.53,0.06) |
| CYC LD |  | **0.23(0.08,0.61)** | **0.45(0.45,0.81)** | **-0.34(-0.53,-0.12)** |
| CYC HD |  | **0.40(0.20,0.74)** | **0.65(0.65,0.89)** | **-0.22(-0.38,-0.07)** |
| LEF HD |  | 0.53(0.13,2.06) | 0.75(0.75,1.24) | -0.15(-0.45,0.15) |
| CYC+AZA |  | 0.36(0.07,1.66) | 0.61(0.61,1.18) | -0.24(-0.53,0.11) |
| MMF-AZA |  | 0.32(0.04,2.38) | 0.56(0.56,1.28) | -0.27(-0.58,0.17) |
| RTX+MMF |  | 0.81(0.30,2.17) | 0.92(0.92,1.26) | -0.05(-0.29,0.15) |
| TAC | AZA | 1.40(0.69,2.95) | 1.17(1.17,1.70) | 0.08(-0.09,0.26) |
| CSA |  | **3.20(1.04,10.19)** | **1.53(1.53,2.39)** | **0.26(0.01,0.47)** |
| PLASMA |  | 0.60(0.15,2.49) | 0.75(0.75,1.57) | -0.12(-0.40,0.22) |
| CYC LD |  | 0.42(0.13,1.23) | 0.59(0.59,1.12) | -0.20(-0.40,0.05) |
| CYC HD |  | 0.73(0.32,1.54) | 0.84(0.84,1.26) | -0.08(-0.26,0.10) |
| LEF HD |  | 0.95(0.22,4.10) | 0.98(0.98,1.76) | -0.01(-0.32,0.31) |
| CYC+AZA |  | 0.65(0.13,3.25) | 0.79(0.79,1.63) | -0.10(-0.40,0.27) |
| MMF-AZA |  | 0.57(0.07,4.58) | 0.73(0.73,1.77) | -0.13(-0.45,0.33) |
| RTX+MMF |  | 1.46(0.45,5.00) | 1.19(1.19,1.93) | 0.09(-0.19,0.35) |
| CSA | TAC | 2.28(0.75,7.14) | 1.31(1.31,1.95) | 0.18(-0.06,0.39) |
| PLASMA |  | 0.43(0.10,1.78) | 0.64(0.64,1.30) | -0.20(-0.49,0.14) |
| CYC LD |  | **0.30(0.09,0.91)** | **0.50(0.50,0.95)** | **-0.28(-0.50,-0.02)** |
| CYC HD |  | 0.52(0.21,1.16) | 0.72(0.72,1.08) | -0.16(-0.36,0.04) |
| LEF HD |  | 0.68(0.15,3.00) | 0.84(0.84,1.49) | -0.09(-0.41,0.24) |
| CYC+AZA |  | 0.47(0.08,2.35) | 0.68(0.68,1.37) | -0.18(-0.49,0.19) |
| MMF-AZA |  | 0.41(0.05,3.25) | 0.62(0.62,1.48) | -0.21(-0.55,0.24) |
| RTX+MMF |  | 1.04(0.32,3.39) | 1.02(1.02,1.57) | 0.01(-0.27,0.26) |
| PLASMA | CSA | **0.19(0.04,0.92)** | **0.49(0.49,0.97)** | **-0.38(-0.66,-0.02)** |
| CYC LD |  | **0.13(0.03,0.52)** | **0.38(0.38,0.76)** | **-0.45(-0.69,-0.15)** |
| CYC HD |  | **0.23(0.06,0.72)** | **0.55(0.55,0.87)** | **-0.33(-0.57,-0.08)** |
| LEF HD |  | 0.30(0.05,1.63) | 0.64(0.64,1.16) | -0.27(-0.61,0.10) |
| CYC+AZA |  | 0.20(0.03,1.28) | 0.51(0.51,1.08) | -0.36(-0.69,0.05) |
| MMF-AZA |  | 0.18(0.02,1.74) | 0.47(0.47,1.16) | -0.38(-0.73,0.10) |
| RTX+MMF |  | 0.46(0.10,1.92) | 0.78(0.78,1.22) | -0.16(-0.47,0.13) |
| CYC LD | PLASMA | 0.70(0.12,3.51) | 0.79(0.79,2.36) | -0.07(-0.43,0.26) |
| CYC HD |  | 1.22(0.27,5.05) | 1.13(1.13,3.00) | 0.04(-0.31,0.34) |
| LEF HD |  | 1.58(0.23,10.49) | 1.29(1.29,3.76) | 0.10(-0.32,0.51) |
| CYC+AZA |  | 1.09(0.14,7.97) | 1.05(1.05,3.30) | 0.02(-0.40,0.45) |
| MMF-AZA |  | 0.96(0.08,10.29) | 0.97(0.97,3.43) | -0.01(-0.45,0.50) |
| RTX+MMF |  | 2.44(0.44,13.48) | 1.58(1.58,4.28) | 0.21(-0.19,0.55) |
| CYC HD | CYC LD | 1.75(0.79,3.94) | 1.43(1.43,2.82) | 0.12(-0.05,0.27) |
| LEF HD |  | 2.29(0.55,10.20) | 1.64(1.64,4.09) | 0.18(-0.12,0.50) |
| CYC+AZA |  | 1.58(0.31,8.09) | 1.34(1.34,3.58) | 0.09(-0.21,0.45) |
| MMF-AZA |  | 1.38(0.17,11.21) | 1.24(1.24,3.82) | 0.06(-0.27,0.51) |
| RTX+MMF |  | 3.49(0.89,15.83) | 2.00(2.00,5.33) | 0.28(-0.03,0.57) |
| LEF HD | CYC HD | 1.31(0.39,4.60) | 1.16(1.16,2.01) | 0.06(-0.19,0.34) |
| CYC+AZA |  | 0.90(0.21,3.69) | 0.94(0.94,1.83) | -0.02(-0.28,0.30) |
| MMF-AZA |  | 0.79(0.11,5.48) | 0.87(0.87,2.05) | -0.05(-0.36,0.37) |
| RTX+MMF |  | 1.99(0.64,7.06) | 1.40(1.40,2.59) | 0.16(-0.10,0.43) |
| CYC+AZA | LEF HD | 0.68(0.10,4.41) | 0.81(0.81,2.32) | -0.09(-0.47,0.33) |
| MMF-AZA |  | 0.60(0.06,5.97) | 0.76(0.76,2.45) | -0.11(-0.53,0.38) |
| RTX+MMF |  | 1.52(0.29,8.86) | 1.21(1.21,3.38) | 0.10(-0.28,0.47) |
| MMF-AZA | CYC+AZA | 0.89(0.23,3.31) | 0.94(0.94,1.91) | -0.02(-0.27,0.26) |
| RTX+MMF |  | 2.24(0.36,14.90) | 1.49(1.49,5.47) | 0.19(-0.23,0.55) |
| RTX+MMF | MMF-AZA | 2.54(0.27,24.86) | 1.61(1.61,9.12) | 0.21(-0.28,0.60) |
|  |  |  |  |  |
| Random-Effect Model | Residual Deviance | 82.2 vs. 77 data points | | |
|  | Deviance Information Criteria | 389.332 | | |
| Fixed-Effect Model | Residual Deviance | 88.8 vs. 77 data points | | |
|  | Deviance Information Criteria | 390.488 | | |

Based on 37 RCTs with 2,697 patients: 35 two-arm trials and 3 three-arm trials

**Significant odds ratios are in bold**

For absolute rates for events used for calculation of risk difference, please see **Appendix 6**

OR, odds ratio; RR, relative risk; RD, risk difference

CYC, cyclophosphamide; MMF, mycophenolate mofetil; CSA, cyclosporine; TAC, tacrolimus; LEF, leflunomide; PRED, prednisone, prednisolone or methylprednisolone; AZA, azathioprine, RTX, rituximab; PLASMA, plasmapharesis

HD, high dose; LD, low dose; when not specified, it indicates standard dose

Only RTX+MMF (LUNAR, Rovin 2012), RTX+CYC (Li 2009a) and MMF+TAC (Bao 2008) were concomitant combination therapy regimens, rest were sequential, i.e., induction and maintenance

Notes: Merged doses for PRED and CYC and comparing only between treatment but not among doses . We did not lose any study but it is a limitation of this analysis

The odds ratios were transformed to relative risk (RR) and risk difference was done to allow ease for interpretation for clinicians and patients
