# Supplementary material for: Comparative effectiveness of immunosuppressive drugs and corticosteroids for lupus nephritis: a systematic review and network meta-analysis
Source: Syst Rev. 2016 Sep 13;5(1):155. doi: 10.1186/s13643-016-0328-z (PMC5020478; doi:10.1186/s13643-016-0328-z)
Supplement: Additional file 6: — Event rates for the outcomes of interest. (DOCX 19 kb) [file 13643_2016_328_MOESM6_ESM.docx]

**Additional File 6. Event rates for the outcomes of interest**

| **Treatments** | **Event rates** |
| --- | --- |
| **Renal remission/response** | |
| **PRED** | 34.57% (23.49%, 45.69%) |
| **CYC** | 55.25% (40.37%, 67.79%) |
| **MMF** | 63.04% (45.62%, 77.17%) |
| **AZA** | 48.48% (30.54%, 65.66%) |
| **TAC** | 56.84% (37.18%, 74.41%) |
| **CSA** | 74.97% (51.52%, 89.92%) |
| **PLASMA** | 36.05% (13.98%, 65.95%) |
| **CYC LD** | 28.14% (10.05%, 54.94%) |
| **CYC HD** | 40.73% (21.25%, 60.82%) |
| **LEF HD** | 47.23% (16.16%, 79.87%) |
| **CYC+AZA** | 37.87% (9.97%, 75.85%) |
| **MMF-AZA** | 34.90% (5.88%, 81.69%) |
| **RTX+MMF** | 57.79% (28.81%, 82.28%) |
| **Renal Relapse/flare** | |
| **PRED** | 48.79% (0.07%, 62.08%) |
| **AZA** | 24.05% (0.15%, 51.36%) |
| **MMF** | 14.26% (0.11%, 38.26%) |
| **CYC** | 15.14% (0.09%, 35.00%) |
| **CSA** | 18.97% (0.19%, 62.44%) |
| **CYC+AZA** | 22.29% (0.20%, 59.93%) |
| **MMF-AZA** | 28.31% (0.32%, 84.05%) |
| **Amenorrhea/ovarian failure** | |
| PRED | 15.23% (5.64%, 29.98%) |
| CYC | 40.63% (24.18%, 59.00%) |
| MMF | 7.93% (1.81%, 25.75%) |
| AZA | 32.15% (5.06%, 79.45%) |
| CYC LD | 60.79% (11.96%, 94.65%) |
| **Bone marrow toxicity** | |
| **MMF** | 7.26% (5.08%, 9.82%) |
| **CYC SD** | 14.54% (7.91%, 23.34%) |
| **AZA SD** | 15.99% (7.21%, 34.37%) |
| **CYC LD** | 29.41% (9.43%, 62.77%) |
| **CYC HD** | 29.60% (14.17%, 54.56%) |
| **RTX+MMF SD** | 21.56% (4.54%, 62.93%) |
