# Supplementary material for: Comparative effectiveness of immunosuppressive drugs and corticosteroids for lupus nephritis: a systematic review and network meta-analysis
Source: Syst Rev. 2016 Sep 13;5(1):155. doi: 10.1186/s13643-016-0328-z (PMC5020478; doi:10.1186/s13643-016-0328-z)
Supplement: Additional file 7: — Sensitivity analysis including partial/complete renal remission only. (DOCX 29 kb) [file 13643_2016_328_MOESM7_ESM.docx]

**Additional File 7. Sensitivity analysis including partial/complete renal remission only**

| **Treatment** | **Reference** | **OR (95% CrI)** | **RR (95% CrI)** | **RD (95% Crl)** |
| --- | --- | --- | --- | --- |
| CYC | PRED | 2.55 (0.72,9.62) | 1.92 (0.78,4.67) | 0.18 (-0.06,0.46) |
| MMF |  | 3.15 (0.82,12.76) | 2.17 (0.86,5.22) | 0.24 (-0.04,0.53) |
| AZA |  | 1.88 (0.50,7.33) | 1.59 (0.58,4.07) | 0.12 (-0.12,0.40) |
| TAC |  | 2.73 (0.67,11.74) | 2.00 (0.73,4.96) | 0.20 (-0.07,0.51) |
| CSA |  | **6.42 (1.45,31.04)** | **2.98 (1.29,6.73)** | **0.41 (0.07,0.68)** |
| PLASMA |  | 1.09 (0.25,4.70) | 1.07 (0.30,2.78) | 0.01 (-0.16,0.33) |
| CYC LD |  | 0.64 (0.10,3.64) | 0.70 (0.13,2.60) | -0.06 (-0.25,0.24) |
| PRED HD |  | 0.23 (0.03,1.61) | 0.27 (0.04,1.46) | -0.14 (-0.30,0.07) |
| CYC HD |  | 1.13 (0.25,4.68) | 1.10 (0.32,3.14) | 0.02 (-0.19,0.29) |
| LEF HD |  | 1.51 (0.18,11.74) | 1.36 (0.22,4.53) | 0.07 (-0.20,0.52) |
| CYC+AZA |  | 1.04 (0.11,9.26) | 1.03 (0.14,4.06) | 0.01 (-0.23,0.47) |
| MMF-AZA |  | 0.92 (0.06,13.39) | 0.94 (0.08,4.48) | -0.01 (-0.25,0.55) |
| RTX+MMF |  | 2.55 (0.38,17.41) | 1.92 (0.45,5.33) | 0.18 (-0.14,0.60) |
| MMF | CYC | 1.24 (0.67,2.30) | 1.13 (0.78,1.62) | 0.05 (-0.09,0.19) |
| AZA |  | 0.75 (0.31,1.71) | 0.83 (0.45,1.41) | -0.06 (-0.26,0.12) |
| TAC |  | 1.06 (0.52,2.32) | 1.03 (0.65,1.62) | 0.01 (-0.14,0.20) |
| CSA |  | 2.50 (0.84,7.81) | 1.53 (0.91,2.77) | 0.21 (-0.04,0.45) |
| PLASMA |  | 0.43 (0.06,2.92) | 0.56 (0.12,1.93) | -0.17 (-0.49,0.24) |
| CYC LD |  | **0.25 (0.06,0.87)** | **0.37 (0.10,0.92)** | **-0.23 (-0.45,-0.03)** |
| PRED HD |  | **0.09 (0.02,0.40)** | **0.14 (0.03,0.54)** | **-0.32 (-0.54,-0.13)** |
| CYC HD |  | **0.45 (0.18,0.94)** | **0.58 (0.26,0.97)** | **-0.16 (-0.34,-0.01)** |
| LEF HD |  | 0.59 (0.10,3.18) | 0.71 (0.16,1.78) | -0.10 (-0.38,0.26) |
| CYC+AZA |  | 0.41 (0.06,2.51) | 0.54 (0.10,1.58) | -0.17 (-0.43,0.21) |
| MMF-AZA |  | 0.36 (0.03,3.96) | 0.49 (0.05,1.88) | -0.18 (-0.47,0.31) |
| RTX+MMF |  | 1.01 (0.23,4.38) | 1.00 (0.33,2.03) | 0.00 (-0.28,0.33) |
| AZA | MMF | 0.60 (0.25,1.39) | 0.74 (0.41,1.23) | -0.11 (-0.31,0.07) |
| TAC |  | 0.86 (0.41,1.90) | 0.92 (0.57,1.44) | -0.04 (-0.20,0.15) |
| CSA |  | 2.02 (0.59,7.29) | 1.36 (0.76,2.63) | 0.16 (-0.12,0.43) |
| PLASMA |  | 0.34 (0.04,2.47) | 0.49 (0.10,1.72) | -0.22 (-0.56,0.20) |
| CYC LD |  | **0.20 (0.05,0.68)** | **0.32 (0.09,0.80)** | **-0.28 (-0.51,-0.08)** |
| PRED HD |  | **0.07 (0.01,0.33)** | **0.13 (0.02,0.48)** | **-0.37 (-0.61,-0.15)** |
| CYC HD |  | **0.36 (0.15,0.71)** | **0.51 (0.25,0.82)** | **-0.21 (-0.39,-0.07)** |
| LEF HD |  | 0.48 (0.08,2.47) | 0.64 (0.14,1.53) | -0.15 (-0.44,0.21) |
| CYC+AZA |  | 0.33 (0.05,1.95) | 0.48 (0.09,1.37) | -0.21 (-0.49,0.15) |
| MMF-AZA |  | 0.29 (0.02,3.12) | 0.43 (0.04,1.65) | -0.23 (-0.54,0.25) |
| RTX+MMF |  | 0.81 (0.21,3.07) | 0.89 (0.32,1.66) | -0.05 (-0.31,0.25) |
| TAC | AZA | 1.43 (0.59,3.85) | 1.24 (0.70,2.34) | 0.08 (-0.11,0.30) |
| CSA |  | 3.37 (0.90,13.69) | 1.86 (0.94,4.12) | 0.28 (-0.02,0.55) |
| PLASMA |  | 0.57 (0.08,4.10) | 0.67 (0.14,2.54) | -0.10 (-0.43,0.30) |
| CYC LD |  | 0.34 (0.08,1.30) | 0.44 (0.12,1.19) | -0.17 (-0.39,0.05) |
| PRED HD |  | **0.12 (0.02,0.62)** | **0.17 (0.03,0.71)** | **-0.25 (-0.48,-0.07)** |
| CYC HD |  | 0.60 (0.22,1.46) | 0.70 (0.31,1.30) | -0.09 (-0.28,0.08) |
| LEF HD |  | 0.80 (0.13,4.54) | 0.86 (0.19,2.31) | -0.04 (-0.32,0.34) |
| CYC+AZA |  | 0.55 (0.08,3.61) | 0.65 (0.12,2.06) | -0.10 (-0.37,0.29) |
| MMF-AZA |  | 0.48 (0.04,5.71) | 0.59 (0.06,2.43) | -0.12 (-0.41,0.38) |
| RTX+MMF |  | 1.35 (0.28,6.77) | 1.20 (0.38,2.83) | 0.06 (-0.23,0.42) |
| CSA | TAC | 2.35 (0.63,8.85) | 1.48 (0.79,3.09) | 0.20 (-0.11,0.47) |
| PLASMA |  | 0.40 (0.05,2.94) | 0.54 (0.11,1.99) | -0.18 (-0.54,0.24) |
| CYC LD |  | **0.24 (0.05,0.89)** | **0.36 (0.09,0.93)** | **-0.24 (-0.51,-0.02)** |
| PRED HD |  | **0.08 (0.01,0.42)** | **0.14 (0.02,0.54)** | **-0.33 (-0.60,-0.12)** |
| CYC HD |  | 0.42 (0.14,1.03) | 0.56 (0.24,1.02) | -0.17 (-0.40,0.01) |
| LEF HD |  | 0.56 (0.08,3.19) | 0.69 (0.15,1.82) | -0.12 (-0.44,0.26) |
| CYC+AZA |  | 0.39 (0.05,2.46) | 0.53 (0.09,1.61) | -0.18 (-0.49,0.20) |
| MMF-AZA |  | 0.34 (0.03,3.87) | 0.48 (0.05,1.91) | -0.19 (-0.52,0.30) |
| RTX+MMF |  | 0.95 (0.19,4.30) | 0.97 (0.31,2.08) | -0.01 (-0.33,0.32) |
| PLASMA | CSA | 0.17 (0.02,1.34) | 0.36 (0.08,1.17) | -0.38 (-0.72,0.07) |
| CYC LD |  | **0.10 (0.02,0.50)** | **0.24 (0.05,0.69)** | **-0.45 (-0.72,-0.14)** |
| PRED HD |  | **0.04 (0.00,0.22)** | **0.09 (0.01,0.40)** | **-0.54 (-0.79,-0.23)** |
| CYC HD |  | **0.18 (0.04,0.64)** | **0.37 (0.13,0.78)** | **-0.37 (-0.64,-0.09)** |
| LEF HD |  | 0.24 (0.03,1.68) | 0.46 (0.09,1.24) | -0.31 (-0.66,0.12) |
| CYC+AZA |  | 0.16 (0.02,1.32) | 0.35 (0.06,1.13) | -0.37 (-0.70,0.06) |
| MMF-AZA |  | 0.15 (0.01,1.98) | 0.32 (0.03,1.29) | -0.38 (-0.73,0.15) |
| RTX+MMF |  | 0.40 (0.06,2.46) | 0.65 (0.18,1.45) | -0.20 (-0.57,0.20) |
| CYC LD | PLASMA | 0.59 (0.06,5.75) | 0.66 (0.10,4.12) | -0.07 (-0.45,0.26) |
| PRED HD |  | 0.21 (0.02,2.36) | 0.26 (0.03,2.10) | -0.15 (-0.51,0.09) |
| CYC HD |  | 1.04 (0.13,7.97) | 1.03 (0.22,5.34) | 0.01 (-0.39,0.32) |
| LEF HD |  | 1.39 (0.10,17.49) | 1.27 (0.17,7.46) | 0.05 (-0.38,0.54) |
| CYC+AZA |  | 0.96 (0.07,13.21) | 0.97 (0.11,6.36) | -0.01 (-0.41,0.48) |
| MMF-AZA |  | 0.86 (0.04,17.59) | 0.89 (0.06,6.76) | -0.02 (-0.43,0.55) |
| RTX+MMF |  | 2.35 (0.22,26.97) | 1.77 (0.34,9.41) | 0.16 (-0.29,0.62) |
| PRED HD | CYC LD | 0.36 (0.05,2.22) | 0.40 (0.07,2.01) | -0.07 (-0.32,0.08) |
| CYC HD |  | 1.77 (0.63,4.91) | 1.58 (0.72,4.07) | 0.07 (-0.08,0.22) |
| LEF HD |  | 2.36 (0.39,15.08) | 1.91 (0.45,7.55) | 0.12 (-0.12,0.51) |
| CYC+AZA |  | 1.63 (0.23,11.29) | 1.47 (0.28,6.36) | 0.06 (-0.18,0.45) |
| MMF-AZA |  | 1.43 (0.12,17.99) | 1.33 (0.15,7.63) | 0.04 (-0.22,0.55) |
| RTX+MMF |  | 4.01 (0.68,27.21) | 2.68 (0.74,11.32) | 0.23 (-0.06,0.60) |
| CYC HD | PRED HD | **4.93 (1.09,24.10)** | **3.97 (1.07,18.50)** | **0.15 (0.01,0.36)** |
| LEF HD |  | 6.65 (0.78,58.70) | 4.75 (0.80,29.29) | 0.20 (-0.02,0.62) |
| CYC+AZA |  | 4.57 (0.48,45.90) | 3.64 (0.52,24.45) | 0.14 (-0.06,0.57) |
| MMF-AZA |  | 3.99 (0.26,65.76) | 3.26 (0.29,27.35) | 0.12 (-0.09,0.66) |
| RTX+MMF |  | **11.18 (1.51,100.40)** | **6.78 (1.37,42.03)** | **0.32 (0.04,0.70)** |
| LEF HD | CYC HD | 1.34 (0.30,6.25) | 1.23 (0.36,3.15) | 0.05 (-0.16,0.40) |
| CYC+AZA |  | 0.93 (0.18,4.85) | 0.94 (0.22,2.72) | -0.01 (-0.22,0.34) |
| MMF-AZA |  | 0.81 (0.08,8.25) | 0.85 (0.10,3.43) | -0.03 (-0.27,0.45) |
| RTX+MMF |  | 2.23 (0.52,11.50) | 1.70 (0.60,4.68) | 0.16 (-0.10,0.51) |
| CYC+AZA | LEF HD | 0.69 (0.07,6.53) | 0.77 (0.13,3.97) | -0.06 (-0.46,0.35) |
| MMF-AZA |  | 0.60 (0.04,9.53) | 0.70 (0.07,4.51) | -0.07 (-0.51,0.44) |
| RTX+MMF |  | 1.68 (0.21,15.64) | 1.38 (0.37,7.01) | 0.10 (-0.33,0.52) |
| MMF-AZA | CYC+AZA | 0.89 (0.18,4.41) | 0.92 (0.22,2.76) | -0.01 (-0.25,0.29) |
| RTX+MMF |  | 2.45 (0.27,25.54) | 1.82 (0.43,11.19) | 0.16 (-0.26,0.57) |
| RTX+MMF | MMF-AZA | 2.79 (0.18,46.76) | 1.99 (0.38,21.16) | 0.17 (-0.35,0.61) |
|  |  |  |  |  |
| Random-Effect Model | Residual Deviance | 65.57 vs. 65 data points | | |
|  | Deviance Information Criteria | 335.147 | | |
| Fixed-Effect Model | Residual Deviance | 76.22 vs. 65 data points | | |
|  | Deviance Information Criteria | 337.972 | | |
| Note: |  |  |  |  |
|  |  |  |  |  |
| Total Patients | 2415 |  |  |  |
| Total Studies | 31 |  |  |  |
| 2-arm | 28 |  |  |  |
| 3-arm | 3 |  |  |  |

Based on 31 RCTs with 2,415 patients: 28 two-arm trials and 3 three-arm trials

**Significant odds ratios are in bold**

For absolute rates for events used for calculation of risk difference, please see **Appendix 6**

OR, odds ratio; RR, relative risk; RD, risk difference

CYC, cyclophosphamide; MMF, mycophenolate mofetil; CSA, cyclosporine; TAC, tacrolimus; LEF, leflunomide; PRED, prednisone, prednisolone or methylprednisolone; AZA, azathioprine, RTX, rituximab; PLASMA, plasmapharesis

HD, high dose; LD, low dose; when not specified, it indicates standard dose

Only RTX+MMF (LUNAR, Rovin 2012), RTX+CYC (Li 2009a) and MMF+TAC (Bao 2008) were concomitant combination therapy regimens, rest were sequential, i.e., induction and maintenance

Notes: Merged doses for PRED and CYC and comparing only between treatment but not among doses . We did not lose any study but it is a limitation of this analysis

The odds ratios were transformed to relative risk (RR) and risk difference was done to allow ease for interpretation for clinicians and patients.
