# Supplementary material for: Comparative effectiveness of immunosuppressive drugs and corticosteroids for lupus nephritis: a systematic review and network meta-analysis
Source: Syst Rev. 2016 Sep 13;5(1):155. doi: 10.1186/s13643-016-0328-z (PMC5020478; doi:10.1186/s13643-016-0328-z)
Supplement: Additional file 8: — Staircase diagrams for key outcomes. (DOCX 26 kb) [file 13643_2016_328_MOESM8_ESM.docx]

**Additional File 8. Staircase diagrams for key outcomes**

A. **Partial/complete renal remission or** renal response

| RTX + MMF |  |  |  |  |  |  |  |  |  |  |  |  |
| --- | --- | --- | --- | --- | --- | --- | --- | --- | --- | --- | --- | --- |
| 2.54 (0.27,24.86) | MMF-AZA |  |  |  |  |  |  |  |  |  |  |  |
| 2.24 (0.36,14.90) | 0.89 (0.23,3.31) | CYC+AZA |  |  |  |  |  |  |  |  |  |  |
| 1.52 (0.29,8.86) | 0.60 (0.06,5.97) | 0.68 (0.10,4.41) | LEF HD |  |  |  |  |  |  |  |  |  |
| 1.99 (0.64,7.06) | 0.79 (0.11,5.48) | 0.90 (0.21,3.69) | 1.31 (0.39,4.60) | CYC HD |  |  |  |  |  |  |  |  |
| 3.49 (0.89,15.83) | 1.38 (0.17,11.21) | 1.58 (0.31,8.09) | 2.29 (0.55,10.20) | 1.75 (0.79,3.94) | CYC LD |  |  |  |  |  |  |  |
| 2.44 (0.44,13.48) | 0.96 (0.08,10.29) | 1.09 (0.14,7.97) | 1.58 (0.23,10.49) | 1.22 (0.27,5.05) | 0.70 (0.12,3.51) | PLASMA |  |  |  |  |  |  |
| 0.46 (0.10,1.92) | 0.18 (0.02,1.74) | 0.20 (0.03,1.28) | 0.30 (0.05,1.63) | **0.23 (0.06,0.72)** | **0.13 (0.03,0.52)** | **0.19 (0.04,0.92)** | CSA |  |  |  |  |  |
| 1.04 (0.32,3.39) | 0.41 (0.05,3.25) | 0.47 (0.08,2.35) | 0.68 (0.15,3.00) | 0.52 (0.21,1.16) | **0.30 (0.09,0.91)** | 0.43 (0.10,1.78) | 2.28 (0.75,7.14) | TAC |  |  |  |  |
| 1.46 (0.45,5.00) | 0.57 (0.07,4.58) | 0.65 (0.13,3.25) | 0.95 (0.22,4.10) | 0.73 (0.32,1.54) | 0.42 (0.13,1.23) | 0.60 (0.15,2.49) | **3.20 (1.04,10.19)** | 1.40 (0.69,2.95) | AZA |  |  |  |
| 0.81 (0.30,2.17) | 0.32 (0.04,2.38) | 0.36 (0.07,1.66) | 0.53 (0.13,2.06) | **0.40 (0.20,0.74)** | **0.23 (0.08,0.61)** | 0.33 (0.08,1.30) | 1.76 (0.63,5.20) | 0.77 (0.42,1.45) | 0.55 (0.28,1.06) | MMF |  |  |
| 1.11 (0.38,3.40) | 0.44 (0.05,3.41) | 0.50 (0.10,2.40) | 0.73 (0.17,2.96) | 0.56 (0.26,1.09) | **0.32 (0.10,0.89)** | 0.46 (0.12,1.71) | 2.44 (0.96,6.63) | 1.07 (0.59,2.01) | 0.76 (0.41,1.42) | 1.38 (0.91,2.21) | CYC |  |
| 2.61 (0.77,9.08) | 1.03 (0.12,8.60) | 1.16 (0.21,6.13) | 1.70 (0.37,7.78) | 1.31 (0.50,3.07) | 0.75 (0.21,2.39) | 1.07 (0.34,3.50) | **5.69 (2.02,17.61)** | **2.51 (1.11,5.76)** | 1.80 (0.80,3.87) | **3.26 (1.57,6.72)** | **2.35 (1.28,4.23)** | PRED |

B. Renal Relapse/flare

| MMF-AZA |  |  |  |  |  |  |
| --- | --- | --- | --- | --- | --- | --- |
| 1.38 (0.20,10.41) | CYC+AZA |  |  |  |  |  |
| 1.70 (0.10,29.93) | 1.22 (0.16,9.62) | CSA |  |  |  |  |
| 2.22 (0.19,25.63) | 1.60 (0.37,6.50) | 1.32 (0.20,7.93) | CYC |  |  |  |
| 2.36 (0.22,26.29) | 1.71 (0.43,6.36) | 1.40 (0.25,7.52) | 1.06 (0.39,2.99) | MMF |  |  |
| 1.25 (0.12,13.86) | 0.91 (0.24,3.37) | 0.74 (0.15,3.60) | 0.56 (0.22,1.50) | 0.53 (0.29,1.00) | AZA |  |
| 0.41 (0.03,5.97) | 0.30 (0.05,1.77) | 0.24 (0.03,1.94) | **0.19 (0.05,0.65)** | **0.17 (0.04,0.77)** | 0.33 (0.08,1.34) | PRED |

C. Amenorrhea/ovarian failure

| CYC LD |  |  |  |  |
| --- | --- | --- | --- | --- |
| 3.20  (0.55,21.24) | AZA |  |  |  |
| **16.35  (1.97,247.70)** | 5.25  (0.78,54.39) | MMF |  |  |
| 2.23  (0.23,23.20) | 0.69  (0.09,5.08) | **0.13  (0.03,0.41)** | CYC |  |
| 9.06  (0.63,121.50) | 2.74  (0.26,28.27) | 0.48  (0.09,3.04) | **3.81  (1.26,14.31)** | PRED |

D. Bone marrow toxicity

| RTX+MMF SD |  |  |  |  |  |
| --- | --- | --- | --- | --- | --- |
| 0.65 (0.08,5.36) | CYC HD |  |  |  |  |
| 0.66 (0.06,7.15) | 1.01 (0.29,3.75) | CYC LD |  |  |  |
| 1.43 (0.18,11.42) | 2.20 (0.56,8.96) | 2.16 (0.42,10.83) | AZA SD |  |  |
| 1.63 (0.26,11.77) | 2.47 (0.77,9.99) | 2.45 (0.51,13.07) | 1.11 (0.39,4.03) | CYC SD |  |
| 3.50 (0.61,23.14) | **5.36 (1.95,18.08)** | **5.31 (1.26,24.65)** | **2.42 (1.01,7.07)** | **2.16 (1.14,4.03)** | MMF |

Legend: The staircase diagrams show odds ratios for each treatment vs. another, and should be read from the top to the bottom and left to right. For example the panel D shows that compared to MMF, CYC HD, CYC LD, AZA SD and CYC SD were associated with 2.16-5.36 times higher odds of bone marrow toxicity.
